# Biosynthesis of Lysosomally Escaped Apoptotic Bodies Inhibits Inflammasome Synthesis in Macrophages

**DOI:** 10.34133/research.0581

**Published:** 2025-01-23

**Authors:** Jiayi Mao, Wenzheng Xia, Yanglin Wu, Minxiong Li, Yun Zhao, Peisong Zhai, Yuguang Zhang, Tao Zan, Wenguo Cui, Xiaoming Sun

**Affiliations:** ^1^ Department of Plastic and Reconstructive Surgery, Shanghai Ninth People’s Hospital, Shanghai Jiao Tong University School of Medicine, Shanghai 200011, P. R. China.; ^2^ Department of Orthopaedics, Shanghai Tenth People’s Hospital, Tongji University School of Medicine, Shanghai 200072, P. R. China.; ^3^ Department of Oral and Maxillofacial-Head & Neck Oncology, Shanghai Ninth People’s Hospital, Shanghai Jiao Tong University School of Medicine, Shanghai, China.; ^4^ Department of Orthopaedics, Shanghai Key Laboratory for Prevention and Treatment of Bone and Joint Diseases, Shanghai Institute of Traumatology and Orthopaedics, Ruijin Hospital, Shanghai Jiao Tong University School of Medicine, Shanghai 200025, P. R. China.

## Abstract

Hyperglycemia and bacterial colonization in diabetic wounds aberrantly activate Nod-like receptor protein 3 (NLRP3) in macrophages, resulting in extensive inflammatory infiltration and impaired wound healing. Targeted suppression of the NLRP3 inflammasome shows promise in reducing macrophage inflammatory disruptions. However, challenges such as drug off-target effects and degradation via lysosomal capture remain during treatment. In this study, engineered apoptotic bodies (BHB-dABs) derived from adipose stem cells loaded with β-hydroxybutyric acid (BHB) were synthesized via biosynthesis. These vesicles target M1-type macrophages, which highly express the folic acid receptor in the inflammatory microenvironment, and facilitate lysosomal escape through 1,2-distearoyl-*sn*-propyltriyl-3-phosphatidylethanolamine–polyethylene glycol functionalization, which may enhance the efficacy of NLRP3 inhibition for managing diabetic wounds. In vitro studies demonstrated the biocompatibility of BHB-dABs, their selective targeting of M1-type macrophages, and their ability to release BHB within the inflammatory microenvironment via folic acid and folic acid receptor signaling. These nanovesicles exhibited lysosomal escape, anti-inflammatory, mitochondrial protection, and endothelial cell vascularization properties. In vivo experiments demonstrated that BHB-dABs enhance the recovery of diabetic wound inflammation and angiogenesis, accelerating wound healing. These functionalized apoptotic bodies efficiently deliver NLRP3 inflammasome inhibitors using a dual strategy of targeting macrophages and promoting lysosomal escape. This approach represents a novel therapeutic strategy for effectively treating chronic diabetic wounds.

## Introduction

Inflammation is a natural biological response to injury or infection and is essential for initiating tissue repair and regeneration [1]. However, an exaggerated inflammatory response can lead to tissue damage and chronic disease. Macrophages are central to controlling inflammation and crucial for maintaining the body’s internal environment homeostasis [2]. In diabetic patients, the hyperglycemic conditions at wound sites provoke local inflammation and bacterial infiltration [3,4]. Elevated blood sugar levels also activate Nod-like receptor protein 3 (NLRP3) in macrophages, causing them to polarize into the M1 phenotype and triggering neutrophil extracellular trap production, thereby exacerbating local inflammation [5]. The NLRP3 inflammasome, an essential sensor protein, is crucial in the development of several diseases, including familial periodic autoinflammatory syndromes, Alzheimer’s disease, type 2 diabetes, and atherosclerosis [6]. Activation of the NLRP3 inflammasome triggers a cascade of intracellular signaling events leading to extensive inflammatory infiltration, oxidative stress, and cellular apoptosis [7]. Therefore, targeting M1 macrophages to prevent NLRP3-mediated immune responses represents an effective strategy to mitigate the harmful effects of inflammatory storms and maintain stability in wound healing.

The NLRP3 inflammasome comprises multiprotein complexes assembled by intracytoplasmic pattern recognition receptors, crucial for both disease development and innate immune response [8]. Cell membrane Toll-like receptors recognize lipopolysaccharides (LPSs) and peptidoglycans, harmful components of microbial pathogens infecting cells. This recognition triggers the activation of nuclear transcription factor-κB, inducing the cell to produce inactive NLRP3 monomers and interleukin-1β precursor (pro-IL-1β). Upon infection or damage, cells release danger-associated molecular patterns, including potassium (K^+^) efflux, reactive oxygen species derived from mitochondria, and calcium (Ca^2+^) efflux. Danger-associated molecular patterns bind to receptors, triggering aggregation of apoptosis-associated speck-like protein containing CARD (ASC), NLRP3, and biologically inactive pro-caspase-1 to form NLRP3 inflammasomes in the cytoplasm. Subsequently, pro-caspase-1 is converted into active caspase-1, which then cleaves pro-IL-1β into mature inflammatory components, including IL-1β and IL-18 [9]. The cleavage of gasdermin D (GSDMD) by the active caspase-1 creates pores in the plasma membrane, allowing the release of inflammatory cytokines (IL-1β and IL-18). This process results in cellular pyroptosis and widespread inflammatory infiltration of the surrounding tissue, highlighting another consequence of inflammasome activation [10]. Consequently, NLRP3 inflammasomes are potential therapeutic targets for various inflammatory diseases due to their pivotal role in initiating inflammation [11]. However, challenges such as targeting drug delivery to macrophages, avoiding lysosomal degradation of internalized drugs, and inhibiting intracellular NLRP3 inflammasome synthesis present substantial obstacles to NLRP3-based therapy [12,13].

To enhance the effectiveness of biologics as therapeutic agents, numerous studies have focused on minimizing off-target effects, strengthening interactions between nanoparticles and cells, improving therapeutic outcomes, and promoting tissue regeneration [14]. Yan et al. [15] demonstrated that dopamine and D1 receptor signaling might regulate inflammation in vivo by inhibiting NLRP3 inflammasomes. However, due to inadequate cellular targeting and rapid dopamine degradation, the medication showed poor lesion retention and low bioavailability. Therefore, there is a critical need for drug delivery technologies that enhance efficacy. Traditional platforms like liposomes, micelles, and nanoparticles have been extensively used, but their poor tissue penetration and potential toxicity limit their utility [16,17]. Extracellular vesicles (EVs), characterized by low immunogenicity, excellent biocompatibility, and high tissue penetration, emerge as promising natural nano-delivery vehicles. Modifying the EV membranes and other techniques can optimize drug delivery efficiency [18,19]. For instance, Liu et al. [20] genetically modified human umbilical cord EVs by attaching a type II collagen-targeting peptide (WYRGRL) to their surface and utilized electroporation to incorporate exogenous miR-223 into these vesicles, enabling targeted RNA delivery to cartilage. This approach markedly enhances drug effectiveness. However, these modified EVs are often trapped in lysosomes, leading to their intracellular degradation. Therefore, there is a critical need for vehicles that can enhance targeted endocytosis by cells and facilitate sustained release of therapeutic agents through lysosomal escape. Researchers are now exploring strategies inspired by bacterial and viral mechanisms, utilizing specific chemical agents such as hemagglutinin, *Listeria monocytogenes* O, ammonium chloride, and polyamidopropylamine for lysosomal escape. Further research is necessary to develop lysosomal escape formulations with minimal immunogenicity and toxicity, modular targeting ligand attachment, and potential for cost-effective mass production [21]. Cell-membrane-camouflaged nanoparticles represent a promising approach for therapeutic applications [22]. Apoptotic bodies (ABs), nanoscale EVs formed during programmed cell death, are distinguished by chromatin condensation, cell shrinkage, and cytoplasmic vesiculation, offering inherent advantages of EVs [23,24]. In addition to these inherent advantages, ABs have the unique advantages of attracting macrophages through “eat me” signals such as phosphatidylserine and apoptosis-related proteins C1QC and C3b, as well as stimulating compensatory proliferation of surrounding cells for apoptosis induction, and play a role in inheriting and executing the intrinsic functions of parent cells [25,26].

In this study, we developed a specialized carrier vesicle using biosynthesis to target M1 macrophages by attaching folic acid (FA) to the membrane surface of ABs, exploiting the folic acid receptor (FAR) overexpressed on M1 macrophages [27,28]. To enhance drug delivery efficiency, we combined the amphipathic ionic lipid 1,2-distearoyl-*sn*-propyltriyl-3-phosphatidylethanolamine (DSPE)–polyethylene glycol (PEG) to facilitate ABs’ escape from lysosomes [29–31]. Currently, conventional methods for drug loading and modification in nanocarriers include thin film hydration, ultrasonication, double emulsion, lyophilization, and electroformation. Ultrasonication, with its simplicity, high efficiency, and precise particle size control, has become an important technique for nanovesicle preparation, particularly suitable for small-scale laboratory production and thermally stable drugs. Initially, ADSC-ABs were produced by inducing adipose stem cells (ADSCs) with staurosporine (STS). Porous ABs (pABs) were then obtained through hypoosmotic treatment, followed by centrifugation to remove cell contents and resuspension in phosphate-buffered saline (PBS). DSPE–PEG–FA-modified ABs (dABs) were created by subjecting ABs to low-frequency intermittent oscillation using an ultrasonic cell crusher. This method of physical drug delivery is safer as it does not cause destruction of cellular proteins. Subsequently, the mixed solution of β-hydroxybutyric acid (BHB) with dABs underwent 10 to 15 extrusions through an Avanti extruder (polycarbonate filters with diameters of 425 and 1,000 nm) to yield BHB-dABs with uniform diameters. BHB, a ketone body metabolite produced during low-energy states, inhibits NLRP3 inflammasomes by blocking K^+^ efflux and decreasing ASC oligomerization and speckle formation. These inflammasomes are crucial immune effectors that modulate the innate immune response [32]. In vitro studies assessed the physical and chemical properties, biocompatibility, drug delivery capabilities, lysosomal escape properties, and inflammasome modulation effects of BHB. Additionally, its ability to enhance wound healing was confirmed in a diabetic rat wound model. The findings demonstrate that BHB-dABs improve BHB utilization through enhanced targeted uptake and lysosomal escape, thereby helping to maintain inflammatory balance in chronic diabetic wounds, promoting neoangiogenesis, and facilitating wound healing.

## Results and Discussion

### Preparation and characterization of BHB-dABs

**Fig. 1.**
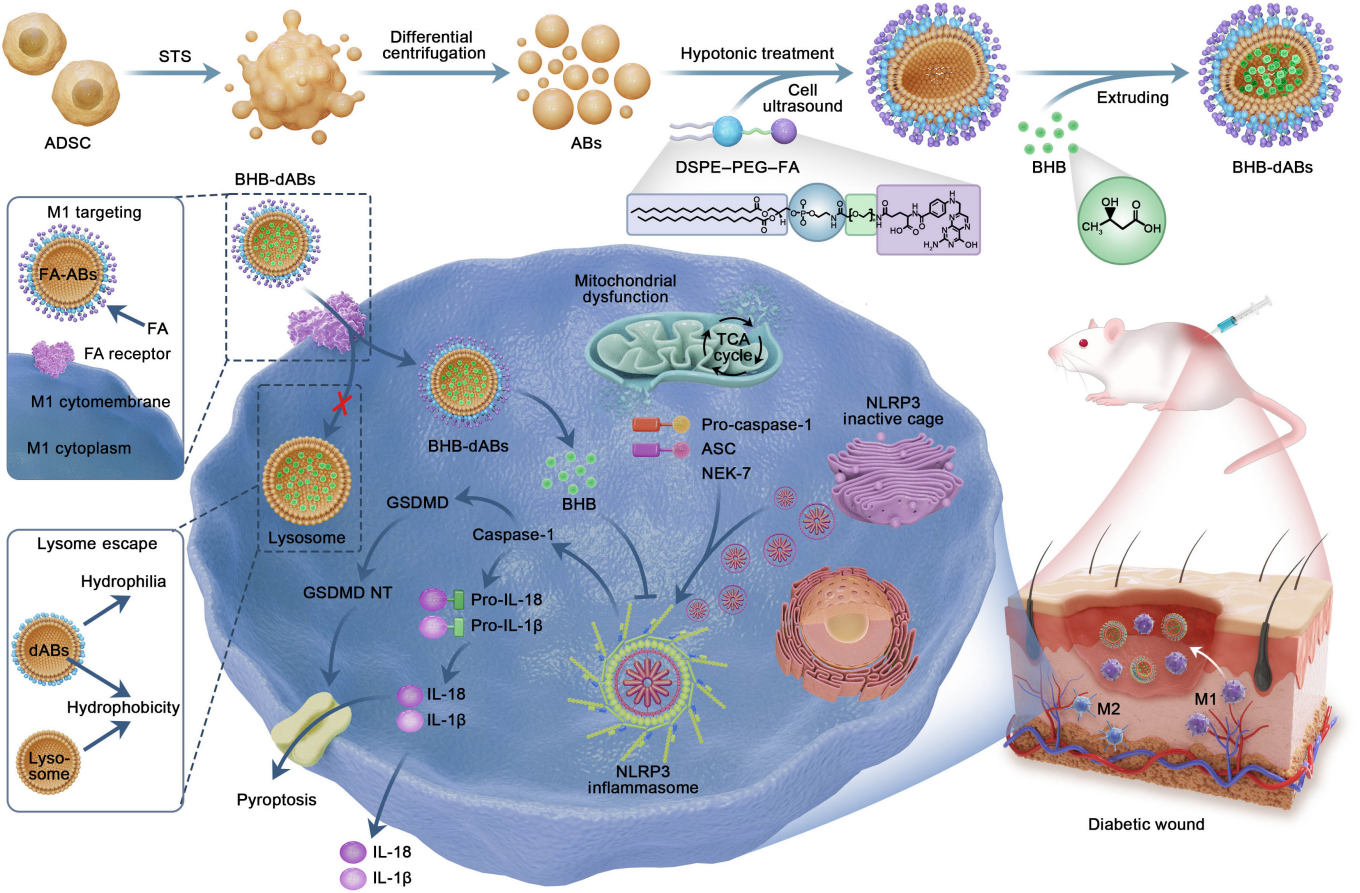
Schematic diagram depicting the preparation of BHB-dABs and their regulation of the Nod-like receptor protein 3 (NLRP3) inflammasome in diabetic wounds. Apoptotic bodies (ABs) were prepared by inducing apoptosis and differential centrifugation of adipose stem cells (ADSCs). Engineered ABs targeted M1 macrophages via ligand–receptor interactions, while lysosomal escape was facilitated by the principle of “like dissolves like”. This dual functionality of the ABs enhanced the pharmacological efficacy of β-hydroxybutyric acid (BHB) and promoted diabetic wound healing. BHB-dABs, DSPE–PEG–FA-modified apoptotic bodies loaded with β-hydroxybutyric acid; DSPE, 1,2-distearoyl-*sn*-propyltriyl-3-phosphatidylethanolamine; PEG, polyethylene glycol; FA, folic acid; STS, staurosporine; ADSC, adipose stem cell; TCA, tricarboxylic acid; ASC, apoptosis-associated speck-like protein containing CARD; NEK-7, NIMA-related kinase 7; GSDMD, gasdermin D; NT, N-terminal fragment; pro-IL-1β, interleukin-1β precursor; IL-1β, interleukin-1β.

To address issues of low drug efficacy related to targeting and lysosomal degradation [33], an improved drug delivery system for ABs was developed based on previous methods [34]. As shown in Fig. 1, apoptosis of ADSCs was triggered by STS to obtain ABs (ADSC-ABs) through differential centrifugation (Fig. S1A and B). These ADSC-ABs retained most components and the intact membrane structure of ADSCs, known for promoting tissue repair by influencing macrophage polarization [35]. dABs loaded with BHB were then prepared using specific techniques, involving hypotonic treatment, cell sonication, and extrusion techniques, to efficiently load BHB, target M1 macrophages, and facilitate lysosomal escape.

**Fig. 2.**
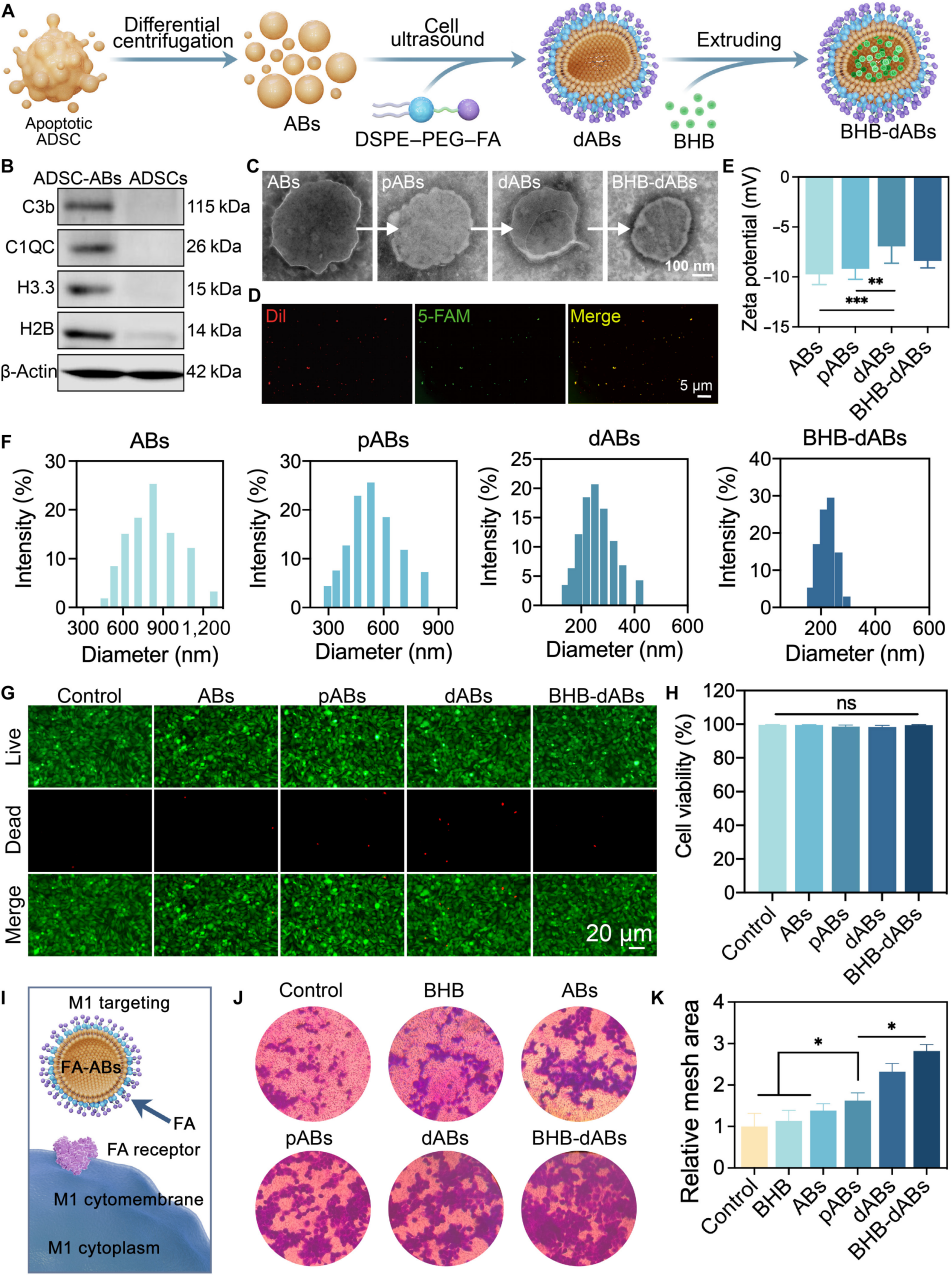
Fabrication and characterization of BHB-dABs. (A) Schematic representation of the fabrication process of BHB-dABs. (B) Typical western blotting images of ADSCs and ADSC-ABs. (C) Typical transmission electron microscopy (TEM) images of ABs during the preparation of BHB-dABs. pABs, porous ABs; dABs, DSPE–PEG–FA-modified apoptotic ABs. (D) Typical immunofluorescence plots of BHB-dABs. 5-FAM, 5-carboxyfluorescein; Dil, 1,1′-dioctadecyl-3,3,3′,3′-tetramethylindocarbocyanine perchlorate. (E) Zeta potential and (F) particle size analysis of different types of ABs. (G) Live/dead staining and (H) statistical analysis of endothelial cells treated with different groups of ABs. (I) Diagram of mechanism for recruitment of M1-type macrophages by BHB-dABs. (J) Typical Transwell plots and (K) statistical analysis of M1-type macrophages recruited by BHB-dABs. ns, *P* > 0.05; *P < 0.05; **P < 0.01; ***P < 0.001.

Firstly, ABs were produced from ADSCs by inducing apoptosis followed by differential centrifugation (Fig. 2A and Fig. S1C). Western blotting was used to characterize the apoptotic proteins in both ADSCs and ADSC-ABs, revealing the expression of apoptotic marker proteins C3b, C1QC, H3.3, and H2B in ABs, while ADSCs did not express these proteins (Fig. 2B). Transmission electron microscopy (TEM) showed that ABs had a bilayer membrane structure with diameters ranging from 400 to 1,000 nm. Upon hypotonic treatment, the surface of pABs exhibited shrinkage and pores. Additionally, the surface characteristics of dABs showed an externally hydrophilic outer membrane layer, while BHB-dABs had a hydrophilic outer membrane layer externally, with BHB loaded internally (Fig. 2C). Fluorescent colocalization staining experiments using 5-carboxyfluorescein (5-FAM)-NH_2_-stained BHB and 1,1′-dioctadecyl-3,3,3′,3′-tetramethylindocarbocyanine perchlorate (Dil)-stained ABs demonstrated successful encapsulation of the drug within ABs using the extrusion technique (Fig. 2D). Subsequently, we analyzed the zeta potential of ABs at various stages. The results indicated no significant change in the membrane potential of pABs and ABs after hypotonic treatment, suggesting that the membrane potential of ABs remained relatively stable following hypotonic treatment. However, the addition of DSPE–PEG significantly decreased the membrane potential of ABs, likely due to neutral molecules encapsulated on the vesicle surface masking some negative charges. The extrusion process might have destroyed part of the neutral molecules’ encapsulation, leading to the exposure of the cell membrane and restoring the exposure of some of the charges (Fig. 2E). The particle size of ABs was assessed by dynamic light scattering. As shown in Fig. 2F, after extrusion treatment, the particle size of ABs decreased and became more uniform, ranging from 180 to 320 nm.

### Drug release profile and biocompatibility of BHB-dABs

The drug loading and release performance of ABs was tested in vitro. BHB-dABs were placed in dialysis bags that had a molecular weight cutoff of 500 Da, following the previous research method [36], ultimately achieving a loading efficiency of 37.60% ± 4.34%. BHB-dABs were periodically evaluated for slow release in PBS at 37 °C. On day 2, approximately 58.04% ± 7.06% of BHB was released from BHB-dABs, and approximately 85.96% ± 4.47% of BHB was released on day 4. Ultimately, drug release lasted ≈6 d (Fig. S1D).

The biocompatibility of ABs in each group was verified through live/dead staining and Cell Counting Kit-8 (CCK-8) cytotoxicity assays. The live/dead staining results showed no significant difference in cell viability among the groups (Fig. 2G and H). Similarly, the CCK-8 assay results revealed no notable differences in the optical density values among the groups (Fig. S1E). These results collectively suggest that BHB-dABs exhibit good biocompatibility.

### Evaluation of M1 macrophage recruitment and lysosomal escape ability

In previous studies targeting M1 macrophages, many researchers have utilized the folate receptor (FAR) on the surface of these cells and designed FA monomer-based targeting moieties to achieve specific modulation of M1 macrophages [27,28]. Here, we performed immunofluorescence staining to investigate the expression of FAR on the surface of M1 macrophages in diabetic wounds. Leukocytes were labeled with CD45, M1 macrophages with CD86, and FAR with PDCR1. The results demonstrated that most CD45^+^CD86^+^ macrophages expressed FAR on their surface, while CD86^−^ cells were predominantly FAR negative, confirming that FAR targeting can achieve macrophage specificity. (Fig. 2I and Fig. S2). The capacity of different ABs to enhance the migration of M1 macrophages was assessed by a Transwell assay. PBS, BHB, ABs, pABs, dABs, and BHB-dABs were placed in the lower chamber, while M1-type macrophages (THP-1 cells were differentiated into M0-type macrophages by phorbol 12-myristate 13-acetate (PMA), and then polarized into M1-type macrophages using LPS + adenosine triphosphate [ATP]) were placed in the upper chamber and co-cultured for 48 h. (The chambers were then taken out and stained with crystal violet [Fig. 2J and K].) The results indicated that the number of macrophages in the AB and pAB groups was significantly higher than in the PBS and BHB groups. This increase is probably attributed to the presence of apoptotic proteins, such as C3b and C1QC, on the membrane surface of ABs and pABs, which have a recruiting effect on macrophages. The synergistic effect of apoptotic proteins and FA ligands on their surface was responsible for the highest macrophage migration observed in the dABs and BHB-dAB groups.

**Fig. 3.**
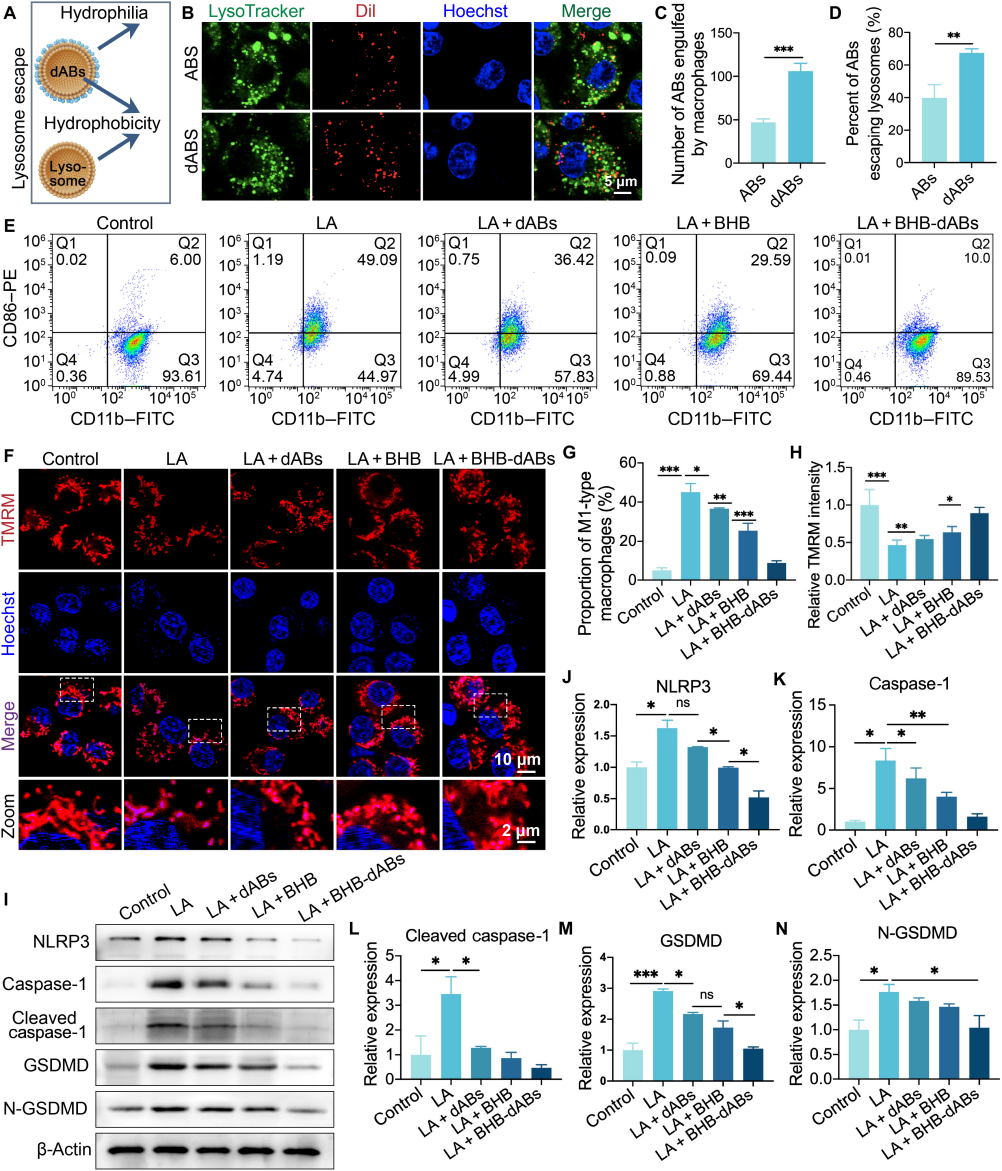
In vitro evaluation of the ability of BHB-dABs to balance inflammation and protect mitochondria. (A) Schematic diagram illustrating the lysosomal escape function of BHB-dABs. (B) Immunofluorescence images showing the lysosomal escape of ABs and dABs after engulfment by macrophages. (C) Statistical analysis of the number of ABs and BHB-dABs phagocytosed by macrophages. (D) Statistical analysis of the lysosomal escape efficiency of ABs and BHB-dABs. (E) Representative flow cytometry plots showing the expression of CD11b and CD86 in THP-1 cells under different stimulation conditions. (F) Typical fluorescence images of the mitochondrial membrane potential of macrophages from different treatment groups for 0, 1, 2, and 3 d. (G) Statistical analysis of the proportion of CD86^+^ cells. (H) Statistical analysis of relative mitochondrial tetramethylrhodamine methyl ester (TMRM) fluorescence intensity. (I) Typical western blotting plots and (J to N) statistical analysis of protein expression in macrophages after different treatments. *P < 0.05; **P < 0.01; ***P < 0.001. PE, phycoerythrin; FITC, fluorescein isothiocyanate; LA, lipopolysaccharide + adenosine triphosphate.

The amphiphilic molecule DSPE–PEG–FA was inserted into the cell membrane using an ultrasonic cell disruptor, facilitated by the hydrophobic interaction between DSPE and the hydrophobic cell membranes. In this process, dABs target macrophages via the ligand–receptor interaction between FA on the dABs and FA receptors on M1 macrophages in vivo, thereby increasing the uptake of ABs by macrophages [37]. Additionally, the hydrophilic PEG is positioned on the outer surface of the cell membrane, forming a hydrophilic outer layer around the originally hydrophobic AB membrane. The endosome–lysosome fusion process is disrupted due to the disruption of similar hydrophobic interactions, which enhances lysosomal escape ability (Fig. 3A). Following previous research methods [38], we observed the colocalization of ABs and THP-1s using a fluorescence microscope. Figure 3B to D demonstrate that THP-1s phagocytosed ABs significantly less than dABs under laser scanning confocal microscopy observation. The number of phagocytosed ABs was approximately 47 ± 4.0, while the number of phagocytosed dABs was approximately 106 ± 8.8, indicating that the phagocytosis efficiency of dABs was 2.25 times that of ABs. In addition, we observed the fate of phagocytosed ABs. The number of ABs that did not colocalize with lysosomes within THP-1 cells after phagocytosis was 18.67 ± 2.78, while the number of dABs was 71.33 ± 3.21. When it came to the phagocytosed particles, ABs and dABs had lysosomal escape rates of 39.83% ± 8.12% and 67.43% ± 2.56%, respectively. According to this, DSPE–PEG modification changes the lipophilic nature of the AB surface to a hydrophilic one, which prevents lysosomal engulfment due to the “like dissolves like” principle.

### Evaluating the capacity of BHB-dABs for macrophage homeostasis, mitochondrial protection, and NLRP3 inflammasome modulation

To further understand the role of BHB-dABs in the M1 polarization of macrophages, we used PMA to differentiate THP-1 cells into M0 macrophages. The polarized macrophages were then cultured under different conditions (PBS, LPS + ATP [LA] + ABs, LA + dABs, LA + BHB, and LA + BHB-dABs) for 24 h. We analyzed changes in the M1 polarization of macrophages using flow cytometry. The results indicated that LPS and ATP stimulation significantly influenced macrophage polarization toward the M1 phenotype when compared to the control group. However, the reduction in macrophage polarization toward the M1 phenotype was observed with both BHB and BHB-dABs, with the protective effect being more pronounced in the BHB-dAB group (Fig. 3E and G).

When excess inflammatory factors are present in the body, mitochondrial function is impaired, leading to a decrease in mitochondrial membrane potential (ΔΨM). ΔΨM (normally positive outside and negative inside, approximately 120 to 180 mV) is generated by the proton pump driven by the reduction potential difference during the electron transport chain when cells respire oxygen, and it is central to many functions of the organelle [39]. Previous studies have demonstrated that elevated levels of BHB can reduce the formation of the NLRP3 inflammasome, counteracting mitochondrial dysfunction triggered by pro-inflammatory cytokines. Additionally, BHB could safeguard mitochondrial function through the down-regulation of acetyl coenzyme A and mitochondrial acetylation, activating citrate synthase, inhibiting fatty acid uptake, and reducing protein acetylation [40]. To validate the beneficial effect of BHB-dABs on mitochondrial function, we followed the methods of previous studies [41] and used the ΔΨM probe tetramethylrhodamine methyl ester (TMRM) for fluorescence microscopy to measure ΔΨM under different treatment conditions (PBS, LA + ABs, LA + dABs, LA + BHB, and LA + BHB-dABs). As illustrated in Fig. 3F, a significant decrease in the fluorescence intensity of mitochondrial TMRM was observed after treatment with LPS and ATP, indicating a reduction in ΔΨM. However, in THP-1s pre-treated with BHB and BHB-dABs followed by inflammatory stimulation, the fluorescence intensity of TMRM markedly increased (Fig. 3H). In addition, the detection results of mitochondrial ATP showed that mitochondrial function was impaired after LPS and ATP treatment; that is, the content of ATP produced by unit cells was significantly reduced, while the ability of cells to produce ATP was restored after BHB-dAB treatment, which further proved that BHB-dABs had a protective effect on macrophage mitochondria (Fig. S4E). This indicates that BHB and BHB-dABs protected mitochondria from damage during inflammatory stimulation. Furthermore, because of their targeted delivery and lysosomal escape, BHB-dABs have a more potent protective effect on mitochondria during NLRP3 inflammasome activation in macrophages.

To determine how BHB-dABs affect inflammasome activation, we used LA (LPS + ATP)-induced THP-1 cells and dABs, BHB, and BHB-dAB-treated LA-induced THP-1 cells, while PBS served as a control. We found that NLRP3 inflammasome-related proteins were significantly increased in THP-1 cells induced by LA, while BHB and BHB-dAB treatment effectively inhibited the activation of NLRP3 inflammasomes and reduced the activation of GSDMD and caspase-1 (Fig. 3I to N and Figs. S3 and S4A to D). These results suggest that the BHB-dAB system protects macrophage function in diabetic wounds.

### Evaluation of the function of BHB-dABs in promoting angiogenesis and fibroblast migration

**Fig. 4.**
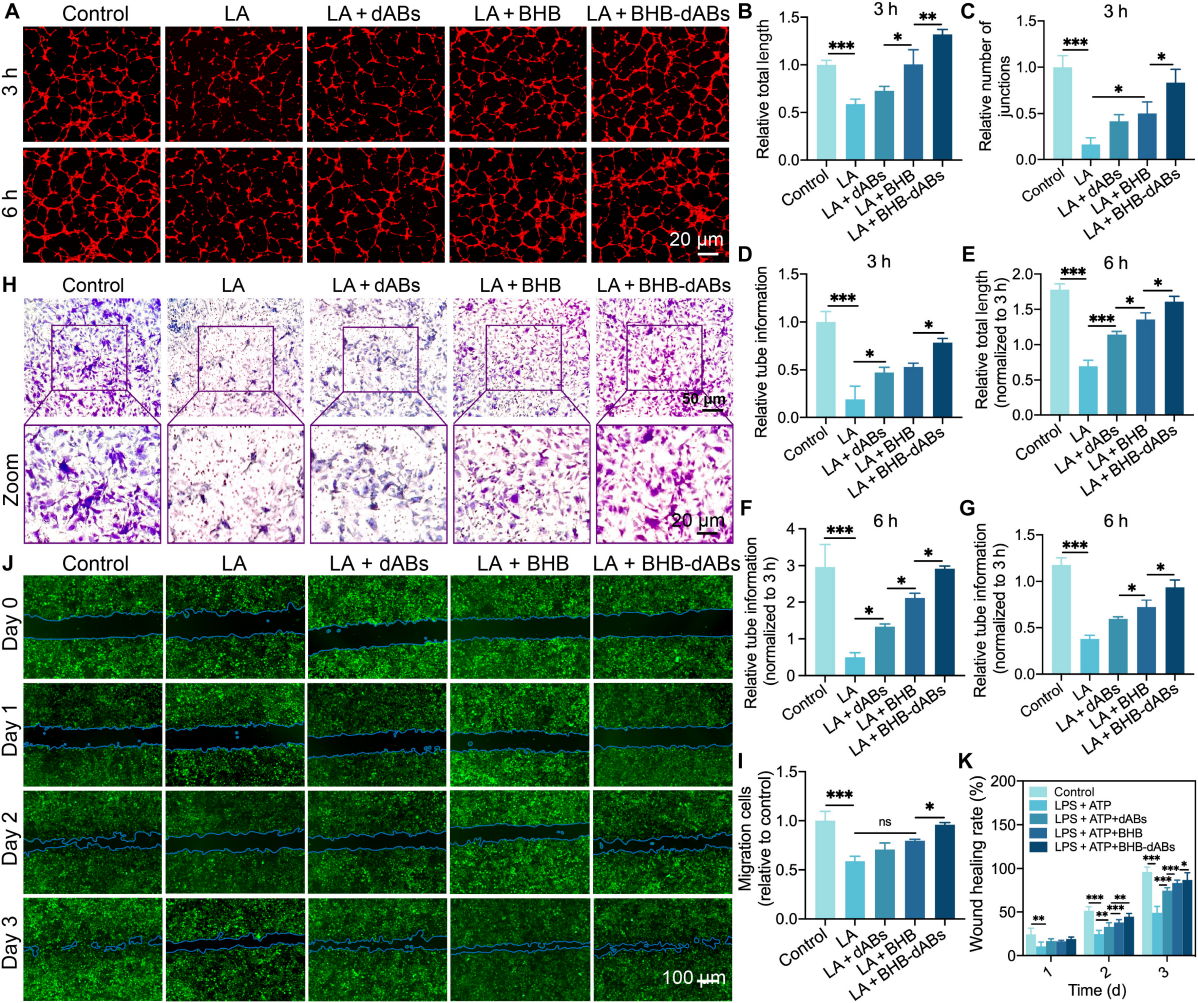
Analysis of the ability of BHB-dABs to promote angiogenesis and to promote fibroblast migration. (A) Typical images of angiogenesis after co-culture of human umbilical vein endothelial cells (HUVECs) with macrophage-conditioned medium treated under different conditions for 3 and 6 h. (B to G) Statistical analysis of the numbers of branching length, junctions, and meshes in tube formation experiments at 3 and 6 h. (H) Representative Transwell migration images of NIH3T3 cells co-cultured with macrophage-conditioned medium treated under different conditions, as detected by crystal violet staining. (I) Statistical analysis of NIH3T3 cell migration. (J) Representative images of the results of a scratch test of HUVECs co-cultured with macrophage-conditioned medium treated under different conditions for 0, 1, 2, and 3 d. (K) Statistical analysis of the results of cell scratching experiments with HUVECs. ns, *P* > 0.05; *P < 0.05; **P < 0.01; ***P < 0.001.

Angiogenesis is crucial in skin repair, as it ensures the delivery of oxygen and nutrients to the damaged area through a sufficient supply of new blood vessels while removing metabolic waste [42,43]. The migration and proliferation abilities of NIH3T3 and human umbilical vein endothelial cells (HUVECs) are also critical factors influencing angiogenesis during wound healing. However, the local microenvironment of diabetic wounds is severely affected by factors such as high glucose levels and extensive inflammation, which markedly impede vascular regeneration in the wound. The results of the angiogenesis experiments in this study (Fig. 4A) demonstrated a significant decrease in vasculogenic ability in the LPS group when compared to that in the control group. This reduction is likely attributable to the adverse effects of the inflammatory environment on angiogenesis. After 3 h, lumen formation was observed in the control group, whereas the LPS group exhibited only scattered nodules or dendritic protrusions (Fig. 4B to D). The BHB and BHB-dAB groups showed significant restoration of endothelial cell vasculogenic ability. The drug’s effective delivery by the carrier vesicles significantly accelerated tube formation, potentially facilitating early vascular formation and wound healing. This trend became more pronounced at 6 h, with almost complete closure of some lumens observed in the control and BHB-dAB groups. Furthermore, the degree of vascular formation was higher than those in other groups (Fig. 4E to G). The enhanced angiogenesis of BHB-dABs is demonstrated by these results in combination with macrophage balancing.

Skin repair involves fibroblasts and endothelial cells migrating as the main components. The effect of BHB-dABs on cell migration was assessed using both Transwell migration assays and scratch assays. Initially, the Transwell migration assay was employed to evaluate the migration ability of NIH3T3 cells in response to conditioned media from macrophages under various conditions. The inflammatory environment had an impact on the growth state of NIH3T3 cells, resulting in poor cell migration in the LPS + ATP (LA) group, which had a negative impact on wound healing. However, the BHB-dAB group showed strong migration activity that was comparable to that of the control group, outperforming the other groups under inflammatory stimulation due to effective inflammation relief (Fig. 4H and I). Furthermore, the scratch assay was performed to evaluate the migration and scratch repair capabilities of HUVECs under various conditions. We obtained conditioned media from THP-1s treated under different conditions and co-cultured them with HUVECs for scratch tests. As shown in Fig. 4J, after 1, 2, and 3 d, the cell migration area increased in all 5 groups of HUVECs. By the third day, the control group achieved a closure area of 96% ± 5.56%, while the closure area for the LA-treated group was only 49% ± 7.20%, and that for the LA + dAB group was 53% ± 4.56%. In contrast, the closure areas of the LA + BHB and LA + BHB-dAB groups reached 83.48% ± 3.26% and 87.01% ± 8.11%, respectively, significantly higher than those of the other 2 groups under inflammatory stimulation (Fig. 4K). This further demonstrates that BHB and BHB-dABs protected macrophages under inflammatory stimulation and promoted endothelial cell migration. This condition is essential for BHB-dABs to promote angiogenesis and healing.

### BHB-dABs substantially improve macrophage function to promote wound healing

**Fig. 5.**
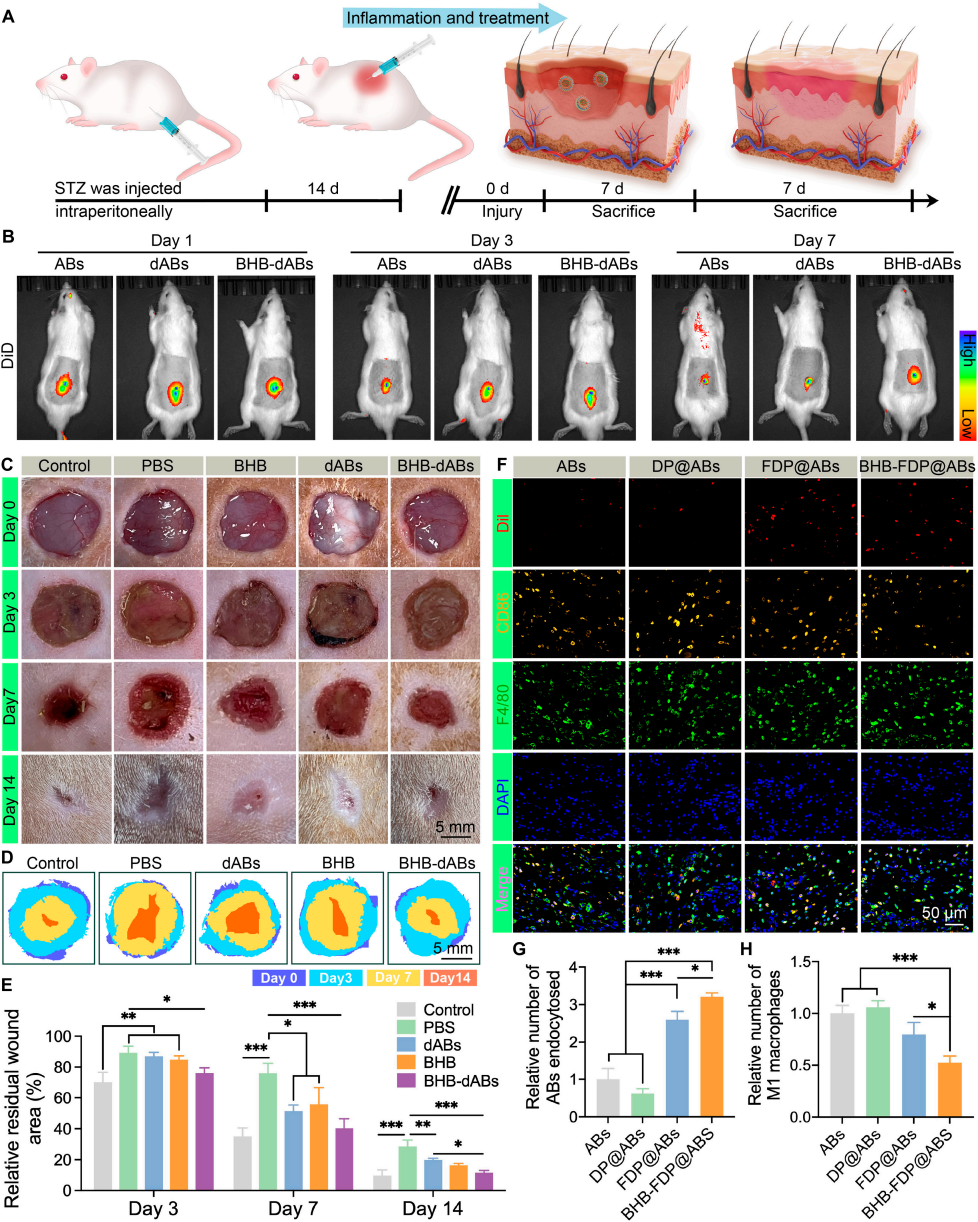
Effect of various interventions on wound healing in vivo. (A) Schematic of diabetic wound model construction and BHB-dAB treatment. (B) In vivo IVIS imaging at various time points. (C) Representative wound images following treatment with control, phosphate-buffered saline (PBS), BHB, dABs, and BHB-dABs at 0, 3, 7, and 14 d. (D) Comparison of wound area size between the various treatment groups. (E) Statistical analysis of wound area sizes across different groups. (F) Typical images and (G and H) statistical analysis of immunofluorescence after treatment with ABs, DP@ABs, FDP@ABs, and BHB-FDP@ABs. *P < 0.05; **P < 0.01; ***P < 0.001. STZ, streptozotocin; DAPI, 4′,6-diamidino-2-phenylindole; DiD, 4′,6-diamidino-2-phenylindole.

Moderate inflammation is beneficial for normal wound healing. However, in pathological conditions like diabetes, excessive inflammation leads to delayed wound closure. This delay is often characterized by persistent pro-inflammatory macrophage polarization [44]. Following the method mentioned by Lin et al. [36], we constructed a rat diabetic wound model (Fig. 5A) and observed the appearance of rat wounds at 0, 3, 7, and 14 d. To evaluate the retention time of agents in the wound, we injected 4′,6-diamidino-2-phenylindole-labeled ABs (DiD-ABs), DiD-dABs, and DiD–BHB-dABs into the rat wound and observed the fluorescence intensity at the defect location using an IVIS Spectrum system. The fluorescence intensity of the defect location of rats injected with DiD-ABs decreased rapidly at 1 week. However, the wounds of rats injected with DiD-dABs and DiD–BHB-dABs showed weak fluorescence at 7 d, suggesting that engineering the dABs with a dual strategy prevented rapid clearance (Fig. 5B).

Compared to wounds on normal skin, diabetic wounds exhibit a substantially slower healing time. In comparison to the PBS group of diabetic rats, the healing process of the 3 treated diabetic wounds was accelerated. Among them, the BHB-dAB group demonstrated a highly effective wound-healing effect by the 14th day, second only to that of the healthy control group of rats (Fig. 5C to E).

The in vivo toxicity of BHB-dABs on major organs was evaluated by hematoxylin and eosin (H&E) staining. No significant damage (e.g., swelling, hemorrhage, congestion, and necrosis) was observed on day 7 of implantation of BHB@dABs compared to the other interventions (control, dABs, and BHB) (Fig. S4), which suggests good biocompatibility of BHB@dABs.

To validate the phagocytosis efficiency of macrophages with different types of ABs, we observed the macrophage phagocytosis using immunofluorescence staining. Fluorescence imaging revealed that CD86^+^ M1-type macrophages with orange fluorescence took up less Dil-stained ABs and DP@ABs, but they phagocytosed significantly more FDP@ABs and BHB-FDP@ABs. Since the FA moiety in FDP (FA–DSPE–PEG) targets M1-type macrophages, the efficiency of targeted delivery is increased (Fig. 5F and G). At 48 h postinjection, the wounds of the ABs-injection and DP@ABs-injection groups exhibited a significantly higher expression of the M1 macrophage marker protein CD86 compared to the FDP@ABs and BHB-FDP@ABs groups. This suggests that FDP@ABs and BHB-FDP@ABs reduced local inflammation by acting on macrophages in the wound 48 h after injection (Fig. 5H). Orange fluorescence labeling CD206 was observed for M2-type macrophages in the tissue. As per the results, M2 macrophages engulfed Dil-stained ABs in a significant way more than DP@ABs, FDP@ABs, and BHB-FDP@ABs. BHB-FDP@ABs led to a substantial rise in M2 macrophages in rats, indicating that BHB-dABs promote the shift of macrophages toward the M2 phenotype (Fig. S5).

### BHB-dABs regulate inflammation, enhance collagen deposition, and stimulate vascular regeneration in vivo

**Fig. 6.**
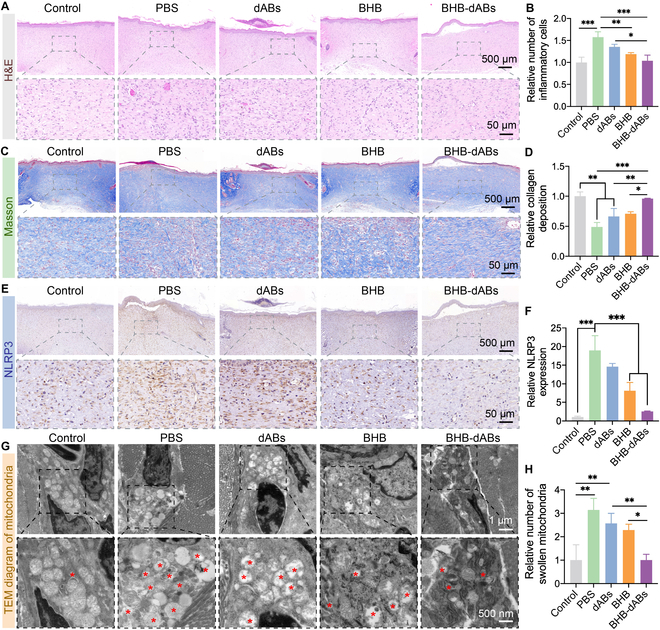
Histological analysis of scar tissues. (A) Representative images and (B) statistical analysis of hematoxylin and eosin (H&E) stains in various groups. (C) Representative images and (D) statistical analysis of Masson’s trichrome staining in different groups. (E) Representative images and (F) statistical analysis of NLRP3 immunohistochemical staining in each group. (G) Representative TEM images and (H) statistical analysis of mitochondrial swelling in each group. *P < 0.05; **P < 0.01; ***P < 0.001.

On day 14, H&E staining analysis was performed on tissue samples collected from the healing skin of rats in each group. As shown in Fig. 6A, H&E staining of the diabetic group showed poorer healing than the control group, with a significant increase in inflammatory cells in the wound. Conversely, the BHB and BHB-dAB groups demonstrated a significant decrease in inflammatory cells. This indicates that BHB alleviated inflammation in the diabetic wound microenvironment, with the delivery of dABs enhancing this effect (Fig. 6B). The analysis of Masson’s trichrome staining showed that the PBS group had significantly lower collagen deposition compared to the control group, whereas all the other 3 treatment groups for diabetes wounds showed improved collagen deposition relative to that of the PBS group, with the BHB-dAB group showing notably higher collagen deposition than the other 2 treatment groups. This suggests that by targeting macrophages and escaping lysosomes, the BHB-dAB system has enhanced BHB delivery capabilities and a faster ability to promote wound healing, as shown in Fig. 6C and D.

To validate BHB’s protection against NLRP3-induced mitochondrial dysfunction, we performed immunohistochemistry staining and TEM imaging on skin specimens from each group of rats. The results indicated that the PBS group had the highest expression levels of NLRP3 and IL-1β, significantly reduced in the BHB and BHB-dAB groups, with the lowest expression of NLRP3 observed in the BHB-dAB group. It is worth noting that the protection and delivery of dABs enhance the efficiency of BHB in inhibiting NLRP3 by 3.18 times and inhibiting IL-1β by 1.94 times compared to that in the drug-only group (Fig. 6E and F and Fig. S6). The TEM results indicate that mitochondria in normal rat wounds have normal morphology, preserved cristae, and no apparent defects. However, in diabetic rat wounds, the outer membrane of the mitochondria is disrupted, leading to fragmentation into numerous circular pieces of varying sizes and a loss of mitochondrial structure. TEM analysis shows that in dABs, the mitochondrial morphology is partially preserved, while in the BHB and BHB-dAB groups, the mitochondrial morphology is mostly preserved. The BHB-dAB group has the least number of swollen mitochondria with disappeared cristae, which indicates that BHB has a protective effect on cellular mitochondria, which is even stronger when dABs are present (Fig. 6G and H).

**Fig. 7.**
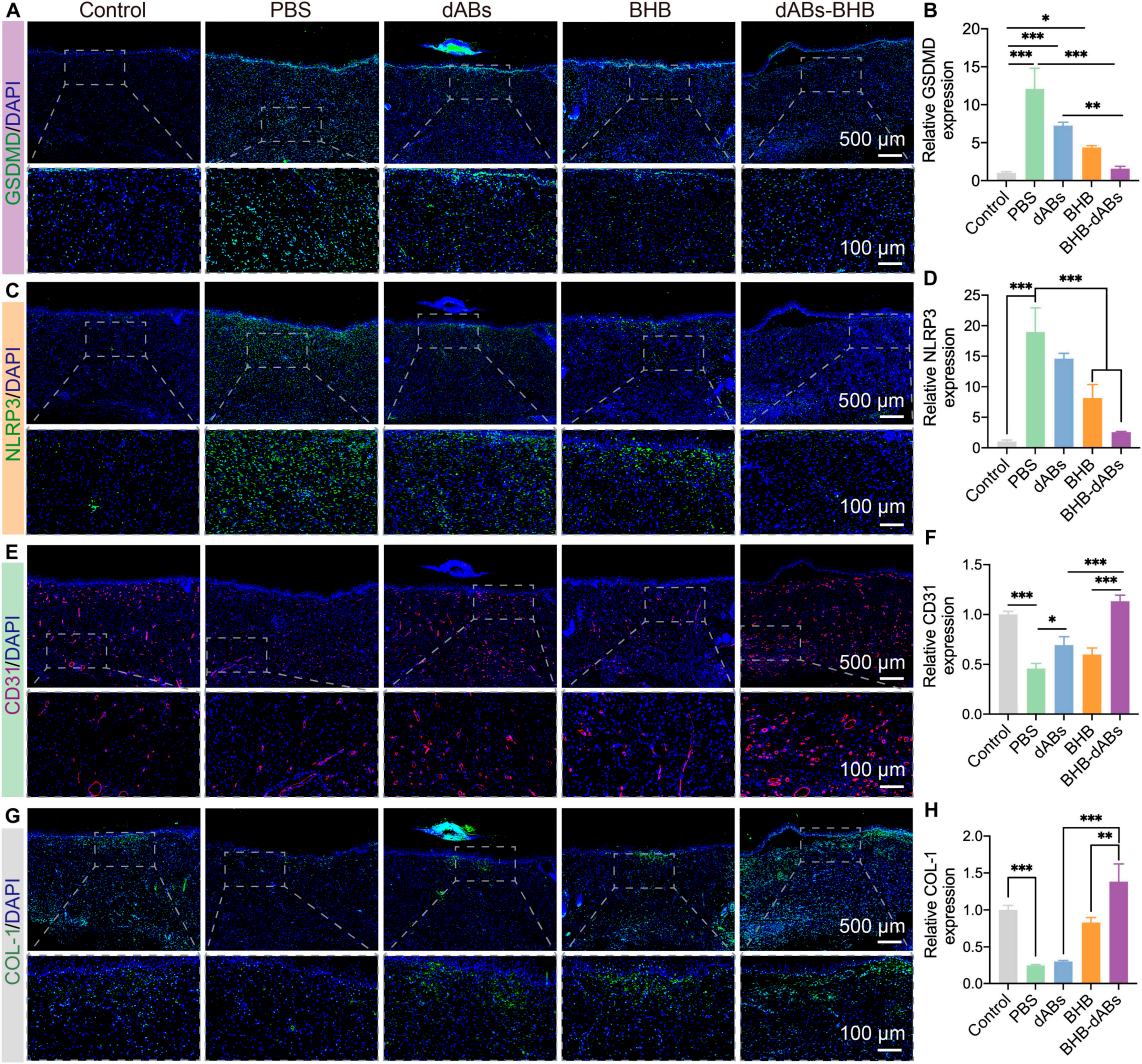
Effects of various treatments on the inflammatory microenvironment, angiogenesis, and collagen deposition in diabetic wounds. (A) Representative images and (B) statistical analysis of GSDMD immunofluorescence staining in each group. (C) Representative images and (D) statistical analysis of NLRP3 immunofluorescence staining in each group. (E) Representative images and (F) statistical analysis of CD31 immunofluorescence staining in each group. (G) Representative images and (H) statistical analysis of COL-1 immunofluorescence staining in each group. *P < 0.05; **P < 0.01; ***P < 0.001.

The accumulation of phenylpyruvate is considerable in diabetic ulcers. After uptake by macrophages, phenylpyruvate interacts with palmitoyl-protein thioesterase 1, inhibiting the activity of the depalmitoylase enzyme, thereby increasing the palmitoylation of NLRP3 protein. This enhances the stability of NLRP3 protein, promotes the activation of NLRP3 inflammasome, and activates caspase-1 and GSDMD, leading to a marked increase in inflammatory factors like IL-1β, ultimately triggering the pro-inflammatory macrophage phenotype [7]. In our study, we employed immunofluorescence staining to assess the expression levels of GSDMD, NLRP3, and IL-1β. The findings indicated that the expression levels of these proteins were significantly higher in diabetic wounds (PBS group) than in normal wounds (control group). The application of dABs in wounds causes reduced GSDMD, NLRP3, and IL-1β in diabetic wounds, suggesting that ABs inheriting the anti-inflammatory effects of ADSCs may be responsible [19,45]. The BHB group effectively inhibited NLRP3 inflammasome activation, resulting in a significant reduction in the expression levels of NLRP3, GSDMD, and IL-1β. BHB-dABs effectively decreased NLRP3 inflammasome activation in macrophages by modulating inflammation via the AB membrane and releasing preloaded drugs in vesicles, which regulate inflammation (Fig. 7A to D and Fig. S7).

To evaluate how different treatments affect angiogenesis and collagen deposition in rat wounds, we performed immunofluorescence staining for endothelial cell marker CD31 and collagen protein COL-1. The results show that on day 14, there was a marked decrease in CD31 expression in the PBS group compared to that in the normal wound group, suggesting a deficiency in microvascular regeneration in diabetic wounds. The diabetic wound group showed an increase in CD31 expression after injection of dABs, BHB, and BHB-dABs into diabetic wounds. CD31 expression did not differ significantly between the dAB and the PBS group. However, the number of new blood vessels in diabetic wounds was significantly increased by BHB, and the BHB-dAB group possessed the highest density of CD31, which was 1.49 times higher than that of the BHB group (Fig. 7E and F). Immunofluorescence staining for collagen protein COL-1 also showed similar results (Fig. 7G and H), further confirming the ability of BHB-dABs to efficiently deliver BHB in the inflammatory microenvironment by targeting M1 macrophages and escaping from lysosomes.

## Conclusion

In this study, we have developed a novel delivery system using nanovesicles, BHB-dABs, which targets macrophages and achieves lysosomal escape simultaneously. By delivering BHB efficiently into cells, this system treats NLRP3 inflammasome overactivation and promotes chronic wound healing. By optimizing hypotonic treatment, cell sonication, and extrusion techniques, we constructed functionalized ABs loaded with BHB and modified with FA–DSPE–PEG. These vesicles target highly FAR-expressing M1 macrophages in the inflammatory microenvironment and achieve lysosomal escape, thereby achieving efficient intracellular delivery of BHB that promotes tissue regeneration and repair. In vitro experiments demonstrated that BHB-dABs were biocompatible, could effectively achieve macrophage recruitment in inflammatory microenvironments and showed the ability to escape lysosomes, successfully inhibited NLRP3 inflammatory vesicles, promoted macrophage conversion to anti-inflammatory phenotypes, and showed excellent protection of endothelial cells and fibroblasts. In vivo experiments demonstrated that BHB-dABs enhance wound healing in diabetic wounds by facilitating the recovery of inflammation and promoting angiogenesis. Therefore, this study shows that these ligand-functionalized dABs enable the efficient release of NLRP3 inhibitors through dual targeting of macrophages and lysosomal escape, providing a new therapeutic strategy for chronic wound healing.

## Methods

### AB extraction process

First, ADSCs (Anwei Biotechnology, Shanghai, China) were cultured in mesenchymal-stem-cell-specific medium at 37 °C with 5% CO_2_. Apoptosis was triggered by introducing 5 μmol/l STS (Sigma-Aldrich, USA) into the medium for 12 h. Cell morphology changes pre- and postapoptosis was observed under light microscopy. Apoptotic cells were harvested using trypsin, while the resulting cell–vesicle suspension was harvested in a 15-ml centrifuge tube and spun at 300 × *g* for 10 min. The supernatant containing apoptotic vesicles was carefully transferred to another 15-ml centrifuge tube, discarding the cellular precipitate. The AB precipitate was obtained through a second centrifugation at 3,000 × *g* for 10 min. The ABs were then resuspended in 1× PBS and stored at −80 °C for subsequent experiments.

### Western blot analysis of surface marker proteins on ABs

ADSCs and ADSC-ABs were lysed using lysis buffer, followed by separate ice-cold lysis at 4 °C for 20 to 30 min. The lysates were subsequently centrifuged at 13,500 × *g* for 15 min. Protein concentrations were measured by Bicinchoninic Acid Protein Assay Kit (abs9232, Absin, Shanghai) and adjusted for consistency. Total proteins were denatured by mixing with sodium dodecyl sulfate-polyacrylamide gel electrophoresis (SDS-PAGE) sampling buffer (P0015, Beyotime, China) and boiling at 100 °C for 10 min. After this step, the proteins were separated via SDS-PAGE and then transferred to polyvinylidene difluoride (PVDF) membranes. The membranes were subsequently blocked for 10 min with block buffer (P0220, Beyotime, China). The membranes were then incubated overnight at 4 °C with following primary antibodies: C3b (21337-1-AP, Proteintech, USA; 1:1,000), C1QC (a675756, Abcam, UK; 1:1,000), H3.3 (ab176840, Abcam, UK; 1:1,000), H2B (ab40886, Abcam, UK; 1:1,000), and β-actin (3700S, CST, USA; 1:1,000). The following day, the PVDF membranes were washed 3 times with Tris-buffered saline with Tween 20 for 10 min each, followed by a 60-min incubation with a secondary antibody (A0208, Beyotime, China; 1:2,000). Ultimately, the visualization of the target proteins was performed using a chemiluminescence detection system (Bio-Rad, Hercules, USA).

### Preparation of BHB-dABs

To start, ABs were initially resuspended in hypotonic lysis buffer containing 10 mmol/l Tris (pH 7.4), 1 mM phenylmethylsulfonyl fluoride, and 10 mM MgCl_2_ for 1 h, 4 °C. This process aimed to achieve pABs, as detailed in previous work [46]. The cells were then centrifuged at 3,000 × *g* for 10 min, after which the supernatant was discarded, followed by resuspension in PBS. This step was repeated twice, with centrifugation at 3,000 × *g* for 10 min each time, to eliminate cellular contents. Subsequently, a combination of ABs, BHB (a concentration of 5 μM was selected based on previous literature reports [34,47]), and DSPE–PEG–FA (PS2-DEFA-2K, Pengshuo Bio, China) was agitated in an ultrasonic cell pulverizer for 10 min at 30-s intervals and a temperature of 25 °C. This process facilitated the insertion of the amphiphilic DSPE–PEG–FA molecules into the cell membranes of the ABs, resulting in the formation of DPF@ABs (dABs), with encapsulated hydrophilic BHB. Centrifugation at 3,000 × *g* was carried out for 10 min at 4°C, the supernatant was discarded, and the pellet was resuspended in PBS, and the procedure was repeated twice to remove superfluous DSPE–PEG–FA and BHB. Finally, BHB-loaded dABs were added to an Avanti extruder (Avanti Polar Lipids Filter Repeat Extruder, Virginia, USA) and passed through polycarbonate filters with pore sizes of 1,000 and 425 nm for 10 to 15 cycles to achieve BHB-dABs uniform in diameter. Care was taken to maintain a slow and consistent extrusion speed to avoid syringe needle clogging.

### Characterization of BHB-dABs

Morphological images of ABs, pABs, dABs, and BHB-dABs were obtained using TEM. Immunofluorescence microscopy was used to confirm the successful encapsulation of BHB by ABs; specifically, ABs were labeled with Dil, and BHB was labeled with 5-FAM-NH_2_ (Xi’an Rui Xi Biotechnology Co., Ltd., Xi’an, China). Since 5-FAM-NH_2_ contains an amino group, it was used to achieve green labeling with the carboxyl group in BHB, and when green fluorescence appeared in red fluorescence, it proved that BHB was successfully loaded into dABs after sonication. Particle diameters and zeta potentials were determined using dynamic light scattering with Zetasizer Nano ZSE (Malvern, UK).

### In vitro drug loading and release evaluation

Following the extrusion of dABs with BHB at a concentration of 5 μM through an extruder, the ABs were then centrifuged and unbound BHB was collected in the supernatant. The concentration was measured using an ultraviolet (UV) spectrophotometer, and the loading efficiency was calculated according to the following equation:

$$\begin{array}{c} \text{Encapsulation efficiency}\;\left(\%\right)=\\ \frac{\text{Amount of total}\;\mathrm{BHB}-\text{Amount of supernatant}\;\mathrm{BHB}}{\text{Amount of total}\;\mathrm{BHB}}\times 100\% \end{array}$$ (1)

For in vitro drug release evaluation, we used UV spectrophotometry. Specifically, 5 μg of BHB-dABs was uniformly dispersed in 2 ml of PBS (1×, pH 7.4) and the mixture was placed in a shaking incubator at 37 °C with a constant speed of 50 rpm. At predetermined time intervals, supernatants were collected from each group via centrifugation, and fresh PBS buffer was added in equal volume. The absorbance of each sample was then measured at a wavelength of 340 nm by an UV spectrophotometer (EV300, Thermo Fisher, Hillsboro, OR, USA).

### Biocompatibility evaluation

On the third day, the evaluation of biocompatibility of ABs, pABs, dABs, and BHB-dABs was conducted using a live/dead cell double staining kit (Sigma-Aldrich, USA) and a CCK-8 assay (Dozindo, Japan). The optical density at 450 nm was measured using a microplate reader (Varioskan Flash 3001, Thermo Scientific, Massachusetts, USA) after adding the CCK-8 reagent to each group (*n* = 5 per group) and incubating for 45 min. The live/dead cell double staining kit was used to stain the cells on day 3 at 37 °C for 30 min, followed by examination under a fluorescence microscope. In vivo toxicity analysis was performed according to the methods of previous studies [48]. H&E histological analysis was performed on major organs (heart, lung, liver, intestine, and kidney) collected from each group of rats. Additionally, cell viability in each group on day 3 was quantified using the ImageJ software.

### Evaluation of macrophage recruitment ability

Human monocytic leukemia cells (THP-1) were treated with 100 ng/ml PMA (Beyotime, S1819, China) for 48 h to promote adhesion and polarization into M0 macrophages and induction of M0 macrophages to M1 macrophages by LPS + ATP (LA). To assess macrophage recruitment, a Transwell migration assay was employed (*n* = 3 per group). A total of 100 μl of M1 macrophage suspensions, with a concentration of 5 × 10^5^ cells/ml, was added to the Transwell chamber. Pure medium, BHB, ABs, dABs, and BHB-dABs were added to the lower chamber containing 600 μl of medium, and the cells were incubated for 48 h. The medium was discarded, and the chambers were removed. The cells were then washed 3 times with PBS and fixed with paraformaldehyde for 20 min. They were subsequently stained with 0.1% crystal violet solution for 20 min and gently rinsed with PBS. The bottom surface of the chamber was examined under an optical microscope, followed by analysis of the results using the ImageJ software.

### Endosome–lysosome escape ability test

A lysosome staining kit was utilized to observe the rapid engulfment and endolysosomal escape of ABs [37]. The colocalization of the endosomal–lysosomal system with apoptotic vesicles was examined in M1-type macrophages induced by LPS and ATP (LPS + ATP, abbreviated as LA) to confirm the phagocytosis and endosomal–lysosomal escape ability of dABs. Macrophages were cultured in confocal culture dishes, and Dil-stained ABs and dABs were then added separately. LysoTracker Green (Beyotime Biotechnology Co, China) was added to the dishes following the kit instructions to stain the lysosomes of macrophages, and 4′,6-diamidino-2-phenylindole (DAPI) was added to stain the cell nuclei. The lysosome escape of macrophages after engulfing ABs was observed using confocal microscopy (Carl Zeiss Ltd., Oberkochen, Germany).

### Flow cytometry analysis of macrophages

Human monocytic leukemia cells (THP-1) were stimulated using 100 ng/ml PMA (Beyotime, S1819, China) for 48 h to induce adhesion and polarization into M0 macrophages. The polarized M0 macrophages were then treated with different conditions (PBS, LA, LA + dABs, LA + BHB, and LA + BHB-dABs). After 24 h, the expression of the macrophage phenotypic markers CD11b (#11-0113-42, eBioscience, USA) and CD86 (#12-2069-42, eBioscience, USA) was analyzed using flow cytometry to analyze the polarization of THP-1 cells toward M1 macrophages.

### Assessment of mitochondrial membrane potential (ΔΨM)

ΔΨM was evaluated by observing macrophages under a laser scanning confocal microscope (Carl Zeiss Ltd., Germany) after exposure to various treatments (PBS, LA [LPS + ATP], LA + dABs, LA + BHB, and LA + BHB-dABs), to study the protective effects of BHB-dABs on ΔΨM damage caused by NLRP3 inflammasome inhibition [39]. The cells were stained with TMRM and Hoechst dyes. First, 80% of the culture medium was aspirated from the fused cells using a pipette. The cells were then washed with 100 μl of PBS. Subsequently, they were incubated at 37 °C with 5% CO_2_ for 45 min in TMRM staining solution (Thermo Scientific, USA) and Hoechst (Thermo Scientific, USA). The cells were washed twice with 100 μl of PBS to eliminate excess TMRM and Hoechst dyes after incubation. The culture dishes containing stained cells were placed on an inverted laser scanning confocal microscope to observe mitochondrial morphology and fluorescence intensity.

### Western blot evaluation

Following treatment of THP-1 (PMA stimulated to M0 macrophages) and bone-marrow-derived macrophages with different conditions (PBS, LA, LA + dABs, LA + BHB, and LA + BHB-dABs), the cells were lysed with radioimmunoprecipitation assay buffer (Beyotime) containing protease and phosphatase inhibitors. The protein concentration was then determined using a bicinchoninic acid assay kit (Beyotime). Subsequently, the supernatant proteins were precipitated using the chloroform–methanol method. After separation by SDS-PAGE, the proteins from the lysed samples were transferred to a PVDF membrane. The membrane was then incubated overnight with the following primary antibodies: anti-NLRP3 antibody (19771-1-Ap, Proteintech, USA; 1:1,000), anti-N-GSDMD antibody (Ab215203, Abcam, UK; 1:500), anti-GSDMD antibody (20770-1-Ap, Proteintech, USA; 1:2,000), anti-cleaved caspase-1 antibody (Af4005, Affinity, USA; 1:1,000), and anti-caspase-1 antibody (Af5418, Affinity, USA; 1:1,000). The PVDF membrane was blocked with a fast block buffer (Beyotime, Shanghai, China) for 30 min at 4 °C. The membrane was then washed 3 times with Tris-buffered saline–Tween and then incubated with a secondary antibody for an hour at room temperature. The results were visualized using a chemiluminescent horseradish peroxidase substrate (ProteinTMorph).

### Evaluation of angiogenesis promoted by BHB-dABs

The study focused on enhancing angiogenesis by BHB-dABs and involved the following steps: Matrigel matrix gel (356234, Corning, USA) was thawed in a refrigerator at 4 °C, and 4 × 10^4^ fluorescein isothiocyanate-labeled HUVECs were inoculated in each well of a 96-well plate. Cell tube formation was monitored using a confocal microscope at different time intervals. The tube formation was analyzed using ImageJ. The impact of conditioned media from macrophages treated with different conditions (PBS, LA, LA + dABs, LA + BHB, and LA + BHB-dABs) on the migration activity of mouse embryonic fibroblasts (NIH3T3) and HUVECs was assessed using Transwell and scratch assays, respectively. NIH3T3 cells were seeded into Transwell chambers (Corning, USA), and conditioned media were subsequently added to the wells of a 24-well plate. The chambers were gently placed into the 24-well plate using forceps. After 24 h, the chambers were removed and cells were fixed with 4% paraformaldehyde. Following this, the cells were stained with crystal violet, and cell migration was assessed with a microscope. HUVEC migration was examined using a scratch assay, where HUVECs were inoculated in 6-well culture plates, and a 200-μl pipette tip was used to create a scratch, followed by washing with PBS, after which conditioned media were added. Images were captured on days 0, 1, 2, and 3. The migration rate was analyzed using the ImageJ software.

### Immunofluorescence staining

Cells were cultured on coverslips, fixed with 4% paraformaldehyde, and permeabilized with Triton X-100, and nonspecific binding was blocked with a serum-based blocking solution. Then, the primary antibody was added and the cells were incubated either overnight at 4 °C or for a short time at room temperature, followed by washing and incubation with a fluorescently labeled secondary antibody in the dark. The nuclei were stained with dyes such as DAPI, and finally, the cells were thoroughly washed with PBS before observation to ensure fluorescence integrity.

### Preparation and treatment of a diabetic rat wound model

The in vivo experimental procedures adhered to the regulations established by the Institutional Animal Care Guidelines. The Animal Research Committee of Shanghai Jiao Tong University approved all procedures. Male Sprague Dawley rats, weighing 180 to 220 g, were fasted for 8 h before the start of the experiments. An intraperitoneal injection of streptozotocin at a dosage of 50 mg/kg was used to induce type 1 diabetes mellitus [49]. A week after confirmation of successful diabetes induction, characterized by blood glucose levels exceeding 16.7 mM and symptoms such as polyphagia, polydipsia, polyuria, and weight loss, diabetic rats were randomly divided into 4 groups: PBS, BHB, dABs, and BHB-dABs, with normal rats serving as controls (*n* = 4 per group). Anesthesia was administered through inhalation of isoflurane, and the back was depilated and shaved using an electric shaver. A circular full-thickness skin wound with a diameter of 10 mm was created on the rats’ backs. Wound-healing progress was monitored on days 1, 3, 7, and 14 using a digital camera. The wound closure rate (CR) was calculated as follows:

$$\mathrm{CR}\;\left(\%\right)=\left(A_{0}-A_{n}\right)/A_{0}\times 100\%$$ (2)

where *A*_0_ is the wound area on day 0 and *A_n_* is the wound area on day *n*.

### In vivo fluorescence imaging

After injection of DiD-ABs, DiD-dABs, and DiD–BHB-dABs into the wounds of diabetic rats, the fluorescence intensity was assessed using an IVIS Spectrum system (Xenogen, USA) at 1, 3, and 7 d.

### In vivo phagocytic efficiency evaluation of BHB-dABs

The efficiency of macrophage phagocytosis of various types of ABs was analyzed using immunofluorescence staining. Initially, the ABs, DSPE–PEG-modified ABs (DP@ABs), FA–DSPE–PEG-modified ABs (FDP@ABs), and FA–DSPE–PEG-modified ABs loaded with BHB (BHB-FDP@ABs) were stained with Dil to label the cell membrane. These stained ABs were subsequently injected subcutaneously, and after 48 h, the tissues were collected, fixed in 4% paraformaldehyde for another 48 h, embedded in paraffin, and sectioned for further analysis. Immunofluorescence staining was performed for the macrophage markers CD86, CD206, and F4/80 and the cell nucleus, with subsequent analysis of fluorescence images using ImageJ.

### Evaluation of mitochondrial morphology in tissues

In accordance with previous research methods [50], tissue samples were initially fixed in 1% osmium tetroxide within 0.1 M sodium cacodylate buffer (pH 7.4) for 1 h at 25 °C. Subsequently, the tissues underwent additional washing in the same buffer and were then dehydrated through a series of graded ethanol solutions. Following dehydration, the samples were embedded in epoxy resin (Epon 812) and polymerized at 60 °C for 48 h. The resulting blocks were sectioned into ultrathin slices with a Leica EM UC7 ultramicrotome, mounted on copper grids, stained with uranyl acetate and lead citrate for contrast, and examined by TEM. For analysis of mitochondrial ultrastructure, a minimum of 3 random images were captured at magnifications ranging from ×5,000 to ×10,000. Parameters such as crista presence, mitochondrial membrane shape, and mitochondrial size were assessed to evaluate mitochondrial morphology.

### Tissue histological evaluation

On the 14th day postwounding, rats were humanely euthanized, and tissue samples from the wound sites were collected. The samples were fixed with 4% paraformaldehyde for 48 h, followed by paraffin embedding and sectioning. Histological analysis included staining with H&E for inflammatory cells, Masson’s trichrome for collagen visualization, and immunohistochemical staining to detect NLRP3 and IL-1β expression. Immunofluorescence staining was also conducted for CD31, COL-1, GSDMD, NLRP3, and IL-1β expression, with quantitative analysis of fluorescence intensity and positive area using the ImageJ software.

### Quantification and statistical assessment

Statistical analyses were performed using the GraphPad Prism software (version 8.0). Data are expressed as mean ± standard deviation, based on 3 or more independent samples. Group differences were evaluated using the *t* test for comparisons between 2 groups. For assessing statistical significance among multiple groups, one-way analysis of variance followed by Tukey’s multiple comparison test was employed. *P* < 0.05 was considered statistically significant (*P < 0.05, **P < 0.01, and ***P < 0.001).

## Data Availability

The datasets used and/or analyzed in the current study are available from the corresponding authors on reasonable request.
