# Supplementary material for: Biosynthesis of Lysosomally Escaped Apoptotic Bodies Inhibits Inflammasome Synthesis in Macrophages
Source: Research (Wash D C). 2025 Jan 23;8:0581. doi: 10.34133/research.0581 (PMC11754539; doi:10.34133/research.0581)
Supplement: Supplementary 1 — Figs. S1 to S8 [file research.0581.f1.zip › Supplementary Information.docx]

**Supplemental Materials**

**Biosynthesis of lysosomally escaped apoptotic vesicles inhibits inflammasome synthesis in macrophages**

Jiayi Mao^1†^, Wenzheng Xia^1†^, Yanglin Wu^3†^, Minxiong Li^1^, Yun Zhao^1^, Peisong Zhai^4^, Yuguang Zhang^1^, Qingfeng Li^1^, Tao Zan^1*^, Wenguo Cui^2*^, Xiaoming Sun^1*^

^1^Department of Plastic and Reconstructive Surgery, Shanghai Ninth People’s Hospital, Shanghai Jiao Tong University School of Medicine, 639 Zhi Zao Ju Road, Shanghai 200011, P. R. China.

^2^Department of Orthopaedics, Shanghai Key Laboratory for Prevention and Treatment of Bone and Joint Diseases, Shanghai Institute of Traumatology and Orthopaedics, Ruijin Hospital, Shanghai Jiao Tong University School of Medicine, 197 Ruijin 2nd Road, Shanghai 200025, P. R. China.

^3^Department of Orthopaedics, Shanghai Tenth People's Hospital, Tongji University School of Medicine, Shanghai, 200072, P. R. China.

^4^Department of Oral and Maxillofacial-Head & Neck Oncology, Shanghai Ninth People's Hospital, Shanghai Jiao Tong University School of Medicine, Shanghai, China.

Address correspondence to: zantao@sjtu.edu.cn (T. Z); wgcui80@hotmail.com (W. C); drsunxm@126.com (X. S).

† These authors contributed equally to this work.

**This file includes:**

Supplementary Figure S1-S6.


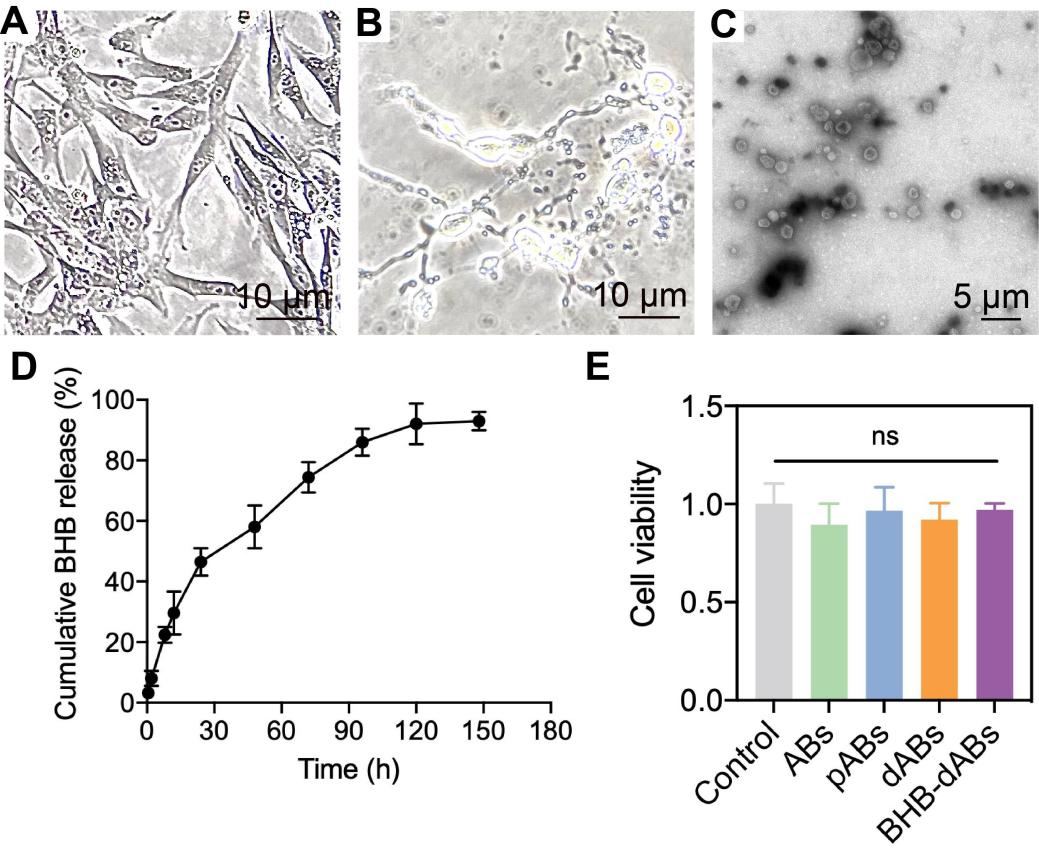


**Figure S1. Extraction and characterization of BHB-dABs. A)** Bright field image of ADSCs. **B)** Bright field image of apoptotic ADSCs after treatment with STS. **C)** Typical TEM images of adipose stem cell-derived apoptotic vesicles (ADSCs-ABs). **D)** In vitro drug release profiles of BHB-dABs. **E)** CCK-8 assay was used to detect the changes in cell viability of endothelial cells treated with different concentrations of ABs (n=5).


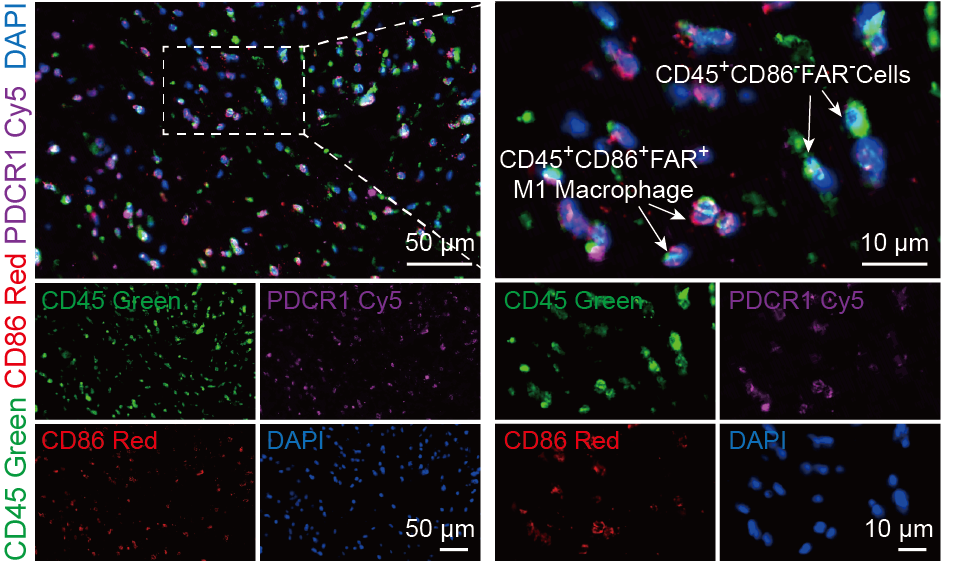


**Figure S2. The FAR receptor on M1 macrophage surface of diabetic wound was detected by immunofluorescence staining.**


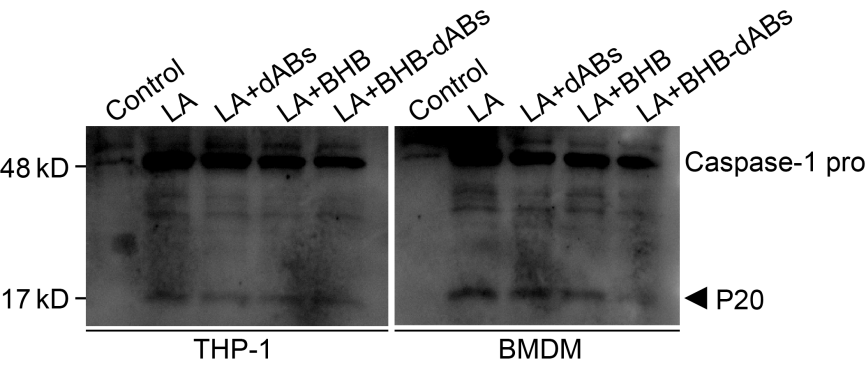


**Figure S3. Western blot results for Caspase-1** Western blot was used to detect the expression of Caspase-1 and its active fragment p-20 in macrophages treated with different concentrations of dABs.

**
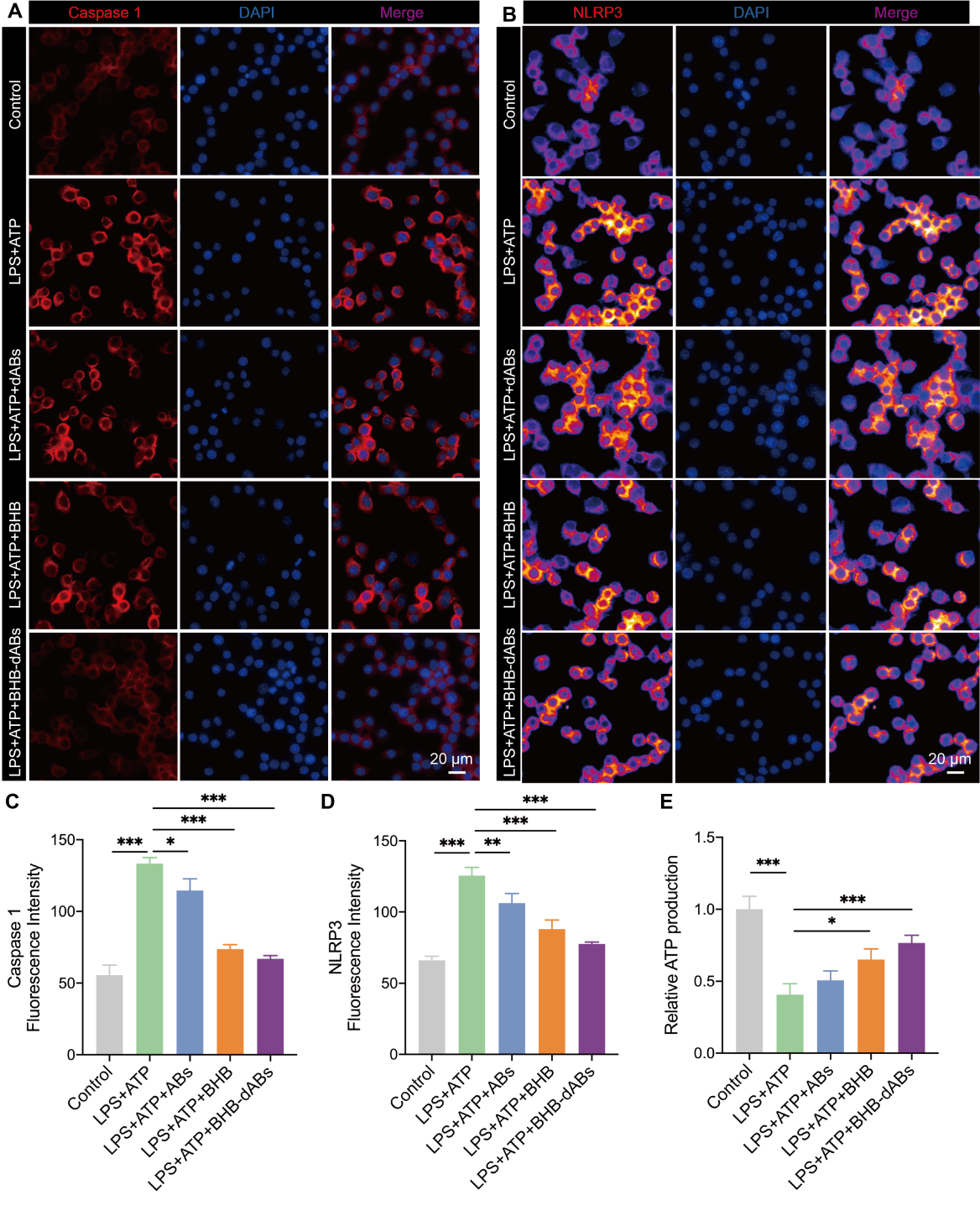
**

**Figure S4. BHB-dABs regulates the macrophage NLRP3 inflammasome and protects mitochondrial function.** A) Immunofluorescence localization of Caspase 1 in macrophages after treatment in different groups. B) Immunofluorescence localization of NLRP3 in macrophages after treatment in different groups. C) Immunofluorescence quantification of Caspase 1 in macrophages after treatment in different groups. D) Immunofluorescence quantification of NLRP3 in macrophages after treatment in different groups. E) ATP production of macrophages after treatment in different groups.


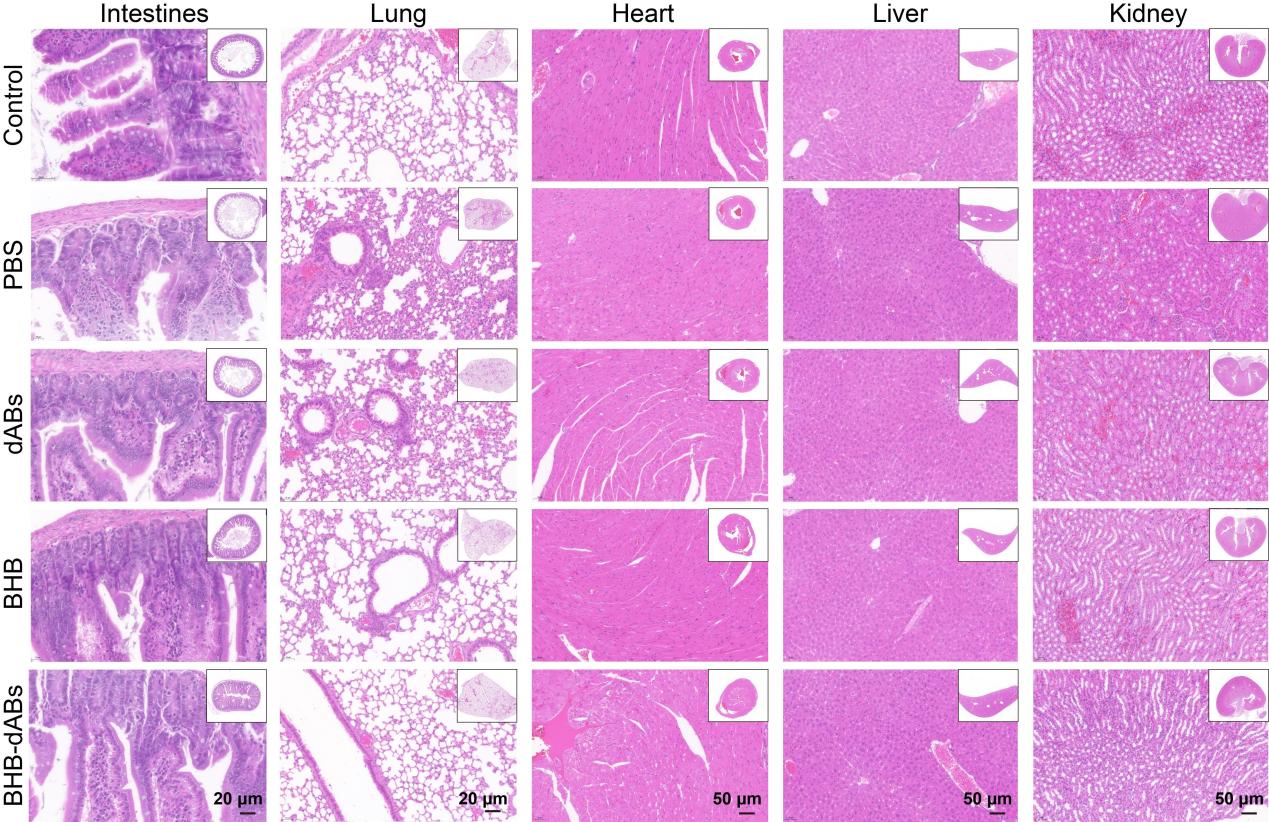


**Figure S5. In vivo biocompatibility evaluation.** H&E staining sections of the major organs (intestine, lung, heart, liver, and kidney) from rats in each group on day 7.


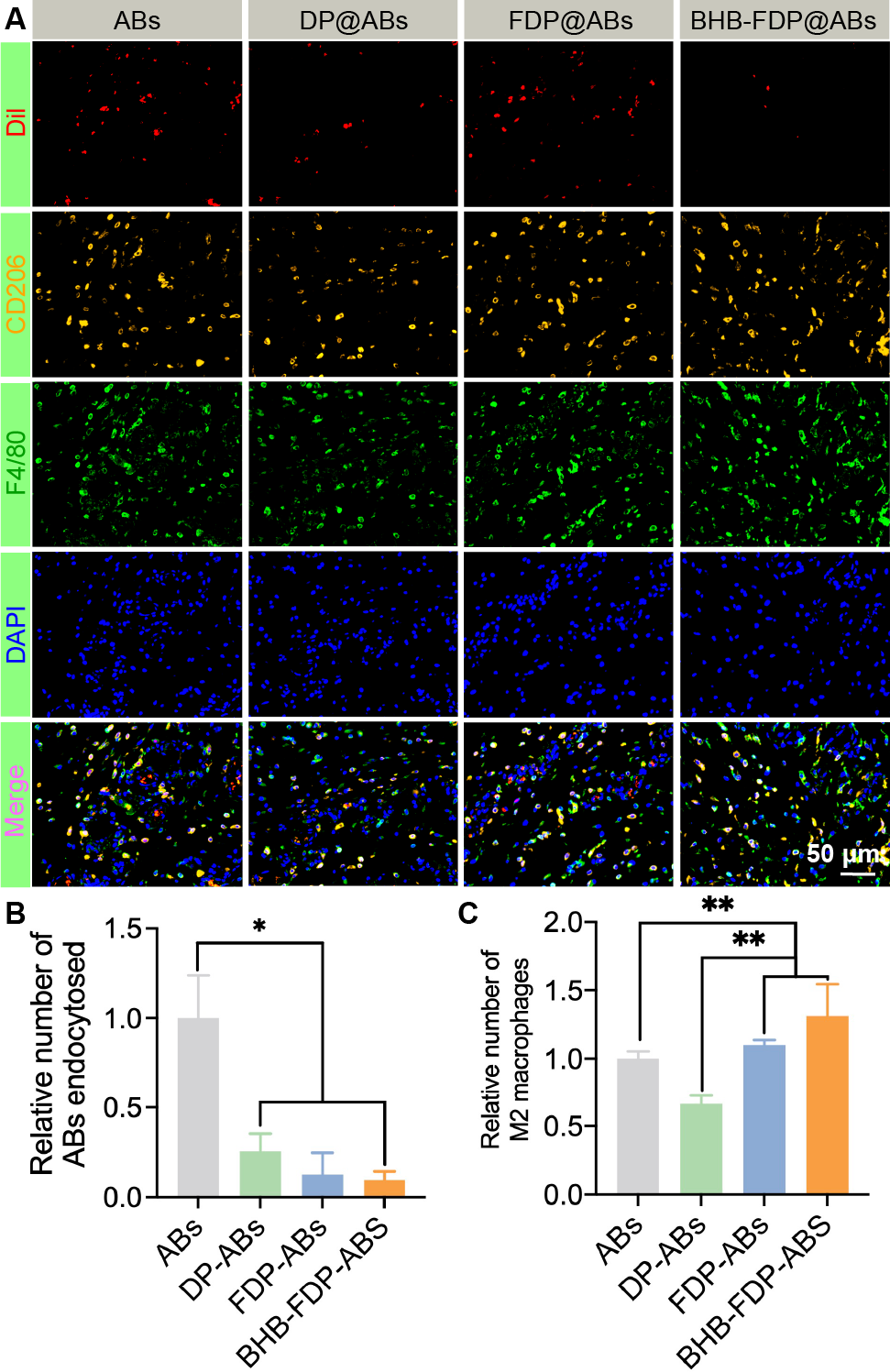


**Figure S6.** **Immunofluorescence staining of M2 macrophages with Dil-ABs. A)** Typical images of post-treatment immunofluorescence of ABs, DP@ABs, FDP@ABs and BHB-FDP@ABs groups. **B)** Statistical analysis of the number of engulfed ABs after treatment in the ABs, DP@ABs, FDP@ABs, and BHB-FDP@ABs groups. **C)** Statistical analysis of the number of M2 macrophages after treatment in the ABs, DP@ABs, FDP@ABs, and BHB-FDP@ABs groups.


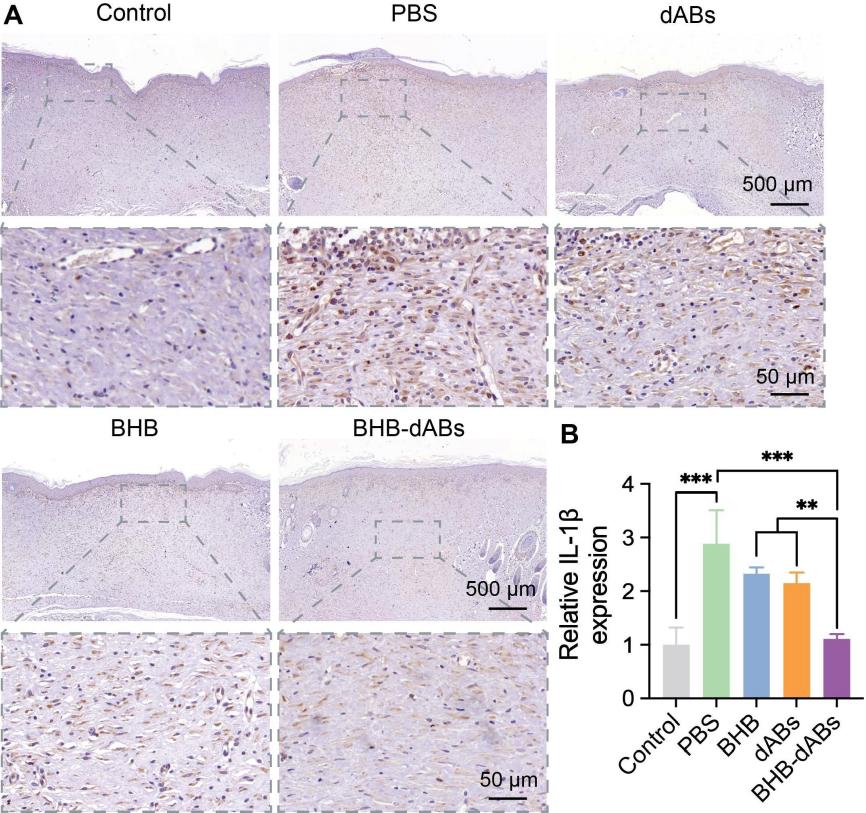


**Figure S7. IL-1β immunohistochemistry of wound tissue. A)** Typical images of IL-1β immunohistochemistry after different groups of treatments. **B)** statistical analysis of IL-1β immunohistochemistry after different groups of treatments.


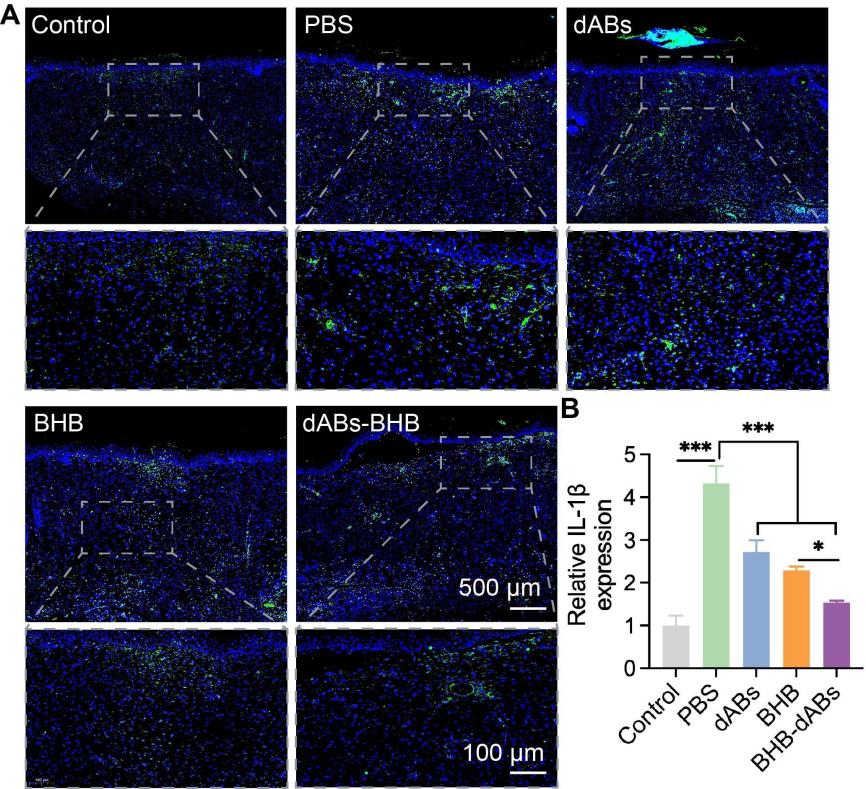


**Figure S8. Immunofluorescence staining of IL-1β in wound tissue. A)** Typical images of IL-1β immunofluorescence staining after different groups of treatments. **B)** statistical analysis of IL-1β immunofluorescence staining after different groups of treatments.
