# Supplementary figures and images for: Biosynthesis of Lysosomally Escaped Apoptotic Bodies Inhibits Inflammasome Synthesis in Macrophages
Source: Research (Wash D C). 2025 Jan 23;8:0581. doi: 10.34133/research.0581 (PMC11754539; doi:10.34133/research.0581)

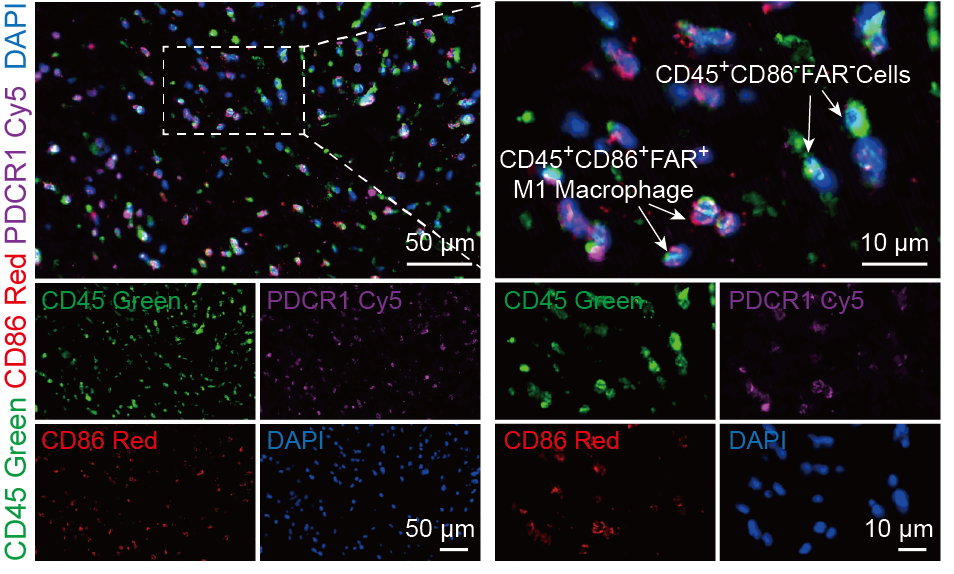

Supplement: Supplementary 1 — Figs. S1 to S8 [file research.0581.f1.zip › S2.tif]

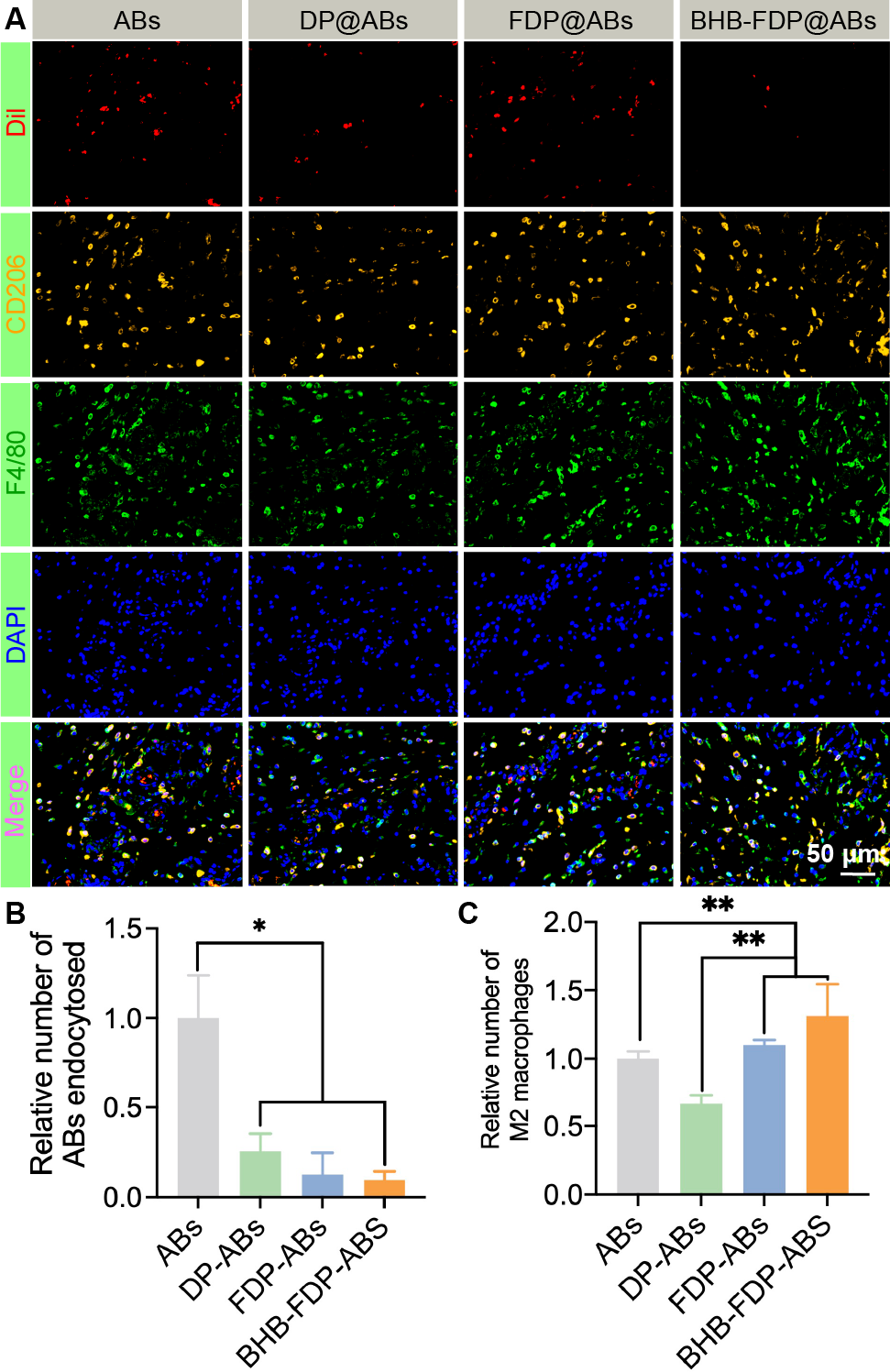

Supplement: Supplementary 1 — Figs. S1 to S8 [file research.0581.f1.zip › S5-cd206.tif]
